# Identification of an Arg-Leu-Arg tripeptide that contributes to the binding interface between the cytokine MIF and the chemokine receptor CXCR4

**DOI:** 10.1038/s41598-018-23554-5

**Published:** 2018-03-26

**Authors:** Michael Lacy, Christos Kontos, Markus Brandhofer, Kathleen Hille, Sabine Gröning, Dzmitry Sinitski, Priscila Bourilhon, Eric Rosenberg, Christine Krammer, Tharshika Thavayogarajah, Georgios Pantouris, Maria Bakou, Christian Weber, Elias Lolis, Jürgen Bernhagen, Aphrodite Kapurniotu

**Affiliations:** 1 Department of Vascular Biology, Institute for Stroke and Dementia Research, Klinikum der Universität München, Ludwig-Maximilians-University of Munich, Feodor-Lynen-Str. 17, D-81377 Munich, Germany; 2 Division of Peptide Biochemistry, Technische Universität München, D-85354 Freising-Weihenstephan, Germany; 3 Department of Anaesthesiology, RWTH Aachen University Hospital, D-52074 Aachen, Germany; 4 Department of Pharmacology, Yale University School of Medicine, 333 Cedar Street, New Haven, CT USA; 5 Institute for Cardiovascular Prevention, Klinikum der Universität München, Ludwig-Maximilians-University of Munich, Pettenkofer Str. 8, D-80336 Munich, Germany; 6 Munich Heart Alliance, D-80802 Munich, Germany; 7 Cardiovascular Research Institute Maastricht, Maastricht University, 6229 Maastricht, The Netherlands; 8 Munich Cluster for Systems Neurology, D-81377 Munich, Germany

## Abstract

MIF is a chemokine-like cytokine that plays a role in the pathogenesis of inflammatory and cardiovascular disorders. It binds to the chemokine-receptors CXCR2/CXCR4 to trigger atherogenic leukocyte migration albeit lacking canonical chemokine structures. We recently characterized an N-like-loop and the Pro-2-residue of MIF as critical molecular determinants of the CXCR4/MIF binding-site and identified allosteric agonism as a mechanism that distinguishes CXCR4-binding to MIF from that to the cognate ligand CXCL12. By using peptide spot-array technology, site-directed mutagenesis, structure-activity-relationships, and molecular docking, we identified the Arg-Leu-Arg (RLR) sequence-region 87–89 that – in three-dimensional space – ‘extends’ the N-like-loop to control site-1-binding to CXCR4. Contrary to wildtype MIF, mutant R87A-L88A-R89A-MIF fails to bind to the N-terminal of CXCR4 and the contribution of RLR to the MIF/CXCR4-interaction is underpinned by an ablation of MIF/CXCR4-specific signaling and reduction in CXCR4-dependent chemotactic leukocyte migration of the RLR-mutant of MIF. Alanine-scanning, functional competition by RLR-containing peptides, and molecular docking indicate that the RLR residues directly participate in contacts between MIF and CXCR4 and highlight the importance of charge-interactions at this interface. Identification of the RLR region adds important structural information to the MIF/CXCR4 binding-site that distinguishes this interface from CXCR4/CXCL12 and will help to design MIF-specific drug-targeting approaches.

## Introduction

Chemokines (CKs) are a complex family of 49 small chemotactic polypeptides, which along with their 23 receptors orchestrate leukocyte migration processes in health and disease. They are structurally characterized by conserved N-terminal cysteine residues and a so-called chemokine-fold and they are sub-divided into four main classes, the CC-, CXC-, C-, and CXXXC-chemokines, based on the nature of the cysteine motif. Chemokine receptors (CKRs) are typical G protein-coupled receptors (GPCRs) with seven transmembrane-spanning α-helices and a C-terminal heterotrimeric G protein-binding domain. CKRs are grouped according to the class of chemokine ligand(s) they interact with. In addition, atypical CKRs (ACKRs) that do not support G_i_ protein-mediated signaling have been defined. Owing to the GPCR nature of chemokine receptors and the involvement of chemokines in numerous pathophysiologic processes they are attractive drug targets^1–4^.

Recent advances in GPCR crystallography have led to the elucidation of the three-dimensional structures of the chemokine receptor CXCR4 complexed to small molecule ligands and the herpesvirus-8 chemokine vMIP-II^5,6^, CCR5 with the FDA-approved compound maraviroc^7^ and a chemokine^8^, CX3CL1 in complex with the human cytomegalovirus GPCR US28^9^, CCR2 in complex with orthosteric and allosteric antagonists^10^, and an intracellular antagonist with CCR9^11^. The structure of CXCR1 has been solved by NMR spectroscopy^12^. Together with structure-activity relationship (SAR) experiments, these studies have helped to understand the activation of chemokine receptors by their cognate ligands and the elicited cellular signaling processes. Most CKs have a two-site mechanism for binding their receptors^13,14^. Site 1 involves interactions between the chemokine N-loop, which follows the N-terminal cysteine motif, and the receptor N-domain. The interactions for site 2 are between the chemokine N-terminal residues prior to the cysteine motif and the extracellular loops (ECLs), e.g. the Glu-Leu-Arg sequence for ELR + chemokines such as CXCL8 in its engagement of CXCR1 or CXCR2^13^. Furthermore, chemokine responses may be fine-tuned by interactions with neighboring glycosaminoglycans (GAGs)^15,16^. Agonist interactions for the homeostatic chemokine receptor CXCR4 are less well understood despite the available X-ray crystallographic information^5,6^. For CXCR4 and its cognate ligand CXCL12 (also known as SDF-1α), the CXCL12 N-loop is comprised of a RFFESH sequence, which interacts with the CXCR4 N-domain (site 1)^17^. CXCL12 lacks an ELR motif and its disordered N-terminus interacts with ECL2 of CXCR4 and penetrates into the transmembrane cavity of the receptor (site 2)^5,13,17^.

Macrophage migration inhibitory factor (MIF) is a multi-functional chemokine-like cytokine that plays a pivotal role in the pathogenesis of numerous inflammatory and cardiovascular disorders such as sepsis, rheumatoid arthritis, systemic lupus erythematosus, inflammatory lung diseases, myocardial ischemia/reperfusion injury, and atherosclerosis^18–22^. It is the prototypical member of an emerging family of mediators with both intra- and extracellular activities termed atypical chemokines (ACKs) or chemokine-like function (CLF) chemokines that, once secreted into the extracellular space, bind to and activate classical chemokine receptors albeit lacking the canonical structural elements^23–25^. Other examples of ACKs are human β-defensin-1 (HBD-1) that binds to CCR6, a secreted tyrosyl tRNA synthetase fragment that is an agonist of CXCR1, or β3-defensin that is an agonist for CXCR4. A complex between the alarmin HMGB1 and CXCL12 also binds to CXCR4^24,26^. Thus, ACKs add significantly to the complexity and redundancy within the CK/CKR network, but also serve to fine-tune the signaling responses and to increase variability in the network. Like their classical chemokine counterparts, ACKs have been recognized as important players in inflammatory and cardiovascular disease^25,27–29^. ACKs are a heterogeneous functional family of proteins and most members do not share structural similarity with each other^24,25^. Accordingly, the structural basis underlying the engagement of chemokine receptors by these mediators is relatively poorly understood and is likely to differ for each member or chemokine-like function. One example of an ACK for which structural information regarding its binding interface with a cognate CKR has been obtained is HBD1, which mimics a charge cluster exposed on the outside of the three-dimensional structure of CCL20/MIP-3α, the cognate ligand of CCR6^27^, but mimicry elements will be different for other ACKs.

MIF exerts its chemokine activities through interactions with the CXC chemokine receptors CXCR2 and CXCR4 to elicit atherogenic leukocyte recruitment. It also binds to the type-II receptor CD74/invariant chain, driving cell-proliferative responses in inflammation and cancer, and to the chemokine scavenger receptor CXCR7/ACKR3^24,25,30–32^. We showed that MIF binds to CXCR2 by a two-site binding mechanism involving an N-like loop and a pseudo-ELR motif within MIF, thus mimicking interactions between CXCR2 and its ELR + ligand CXCL8^30,33,34^. In contrast, the interaction between MIF and CXCR4 remains incompletely understood. We recently characterized an extended N-like loop and the evolutionarily conserved Pro-2 residue of MIF to constitute critical molecular determinants of the CXCR4/MIF binding site and identified partial allosteric agonism as the mechanism that distinguishes CXCR4 binding to MIF from that to the cognate ligand CXCL12^35^. Yet, due to the lack of a RFFESH motif in MIF and its conformationally constrained N-terminus^36^, this only partly explains the affinity and specificity observed for the MIF/CXCR4 interaction^24,25,30^.

Here, we applied peptide spot array technology, site-directed mutagenesis, structure-activity relationship studies, and molecular docking to identify a discontinuous three-amino acid stretch (Arg-Leu-Arg; RLR) that is remotely located at the C-terminal end of the second α-helix in MIF which may serve to extend the N-like loop of MIF and contribute to site 1 binding to CXCR4. The contribution of these residues to functional MIF/CXCR4 interactions was tested using a CXCR4-specific yeast-based cellular signaling system^37^ and a MIF/CXCR4-dependent chemotactic leukocyte migration assay. We also performed alanine scanning and molecular docking techniques to understand the structural details as well as investigate if the RLR motif directly takes part in site 1 contacts between MIF and CXCR4.

## Results

### Identification of the RLR residues, expression and biochemical characterization of the MIF R87A-L88A-R89A triple mutant

We previously showed that an extended N-like loop sequence in MIF contributes to site 1 binding with CXCR4^35^. The N-like loop comprises a flexible loop region followed by residues of the ensuing β-strand. Residues at the C-terminal of this region have been implied in binding to MIF receptor CD74 (amino acids 79–86)^38^, but not in interactions between MIF and CXCR4.

**Figure 1:**
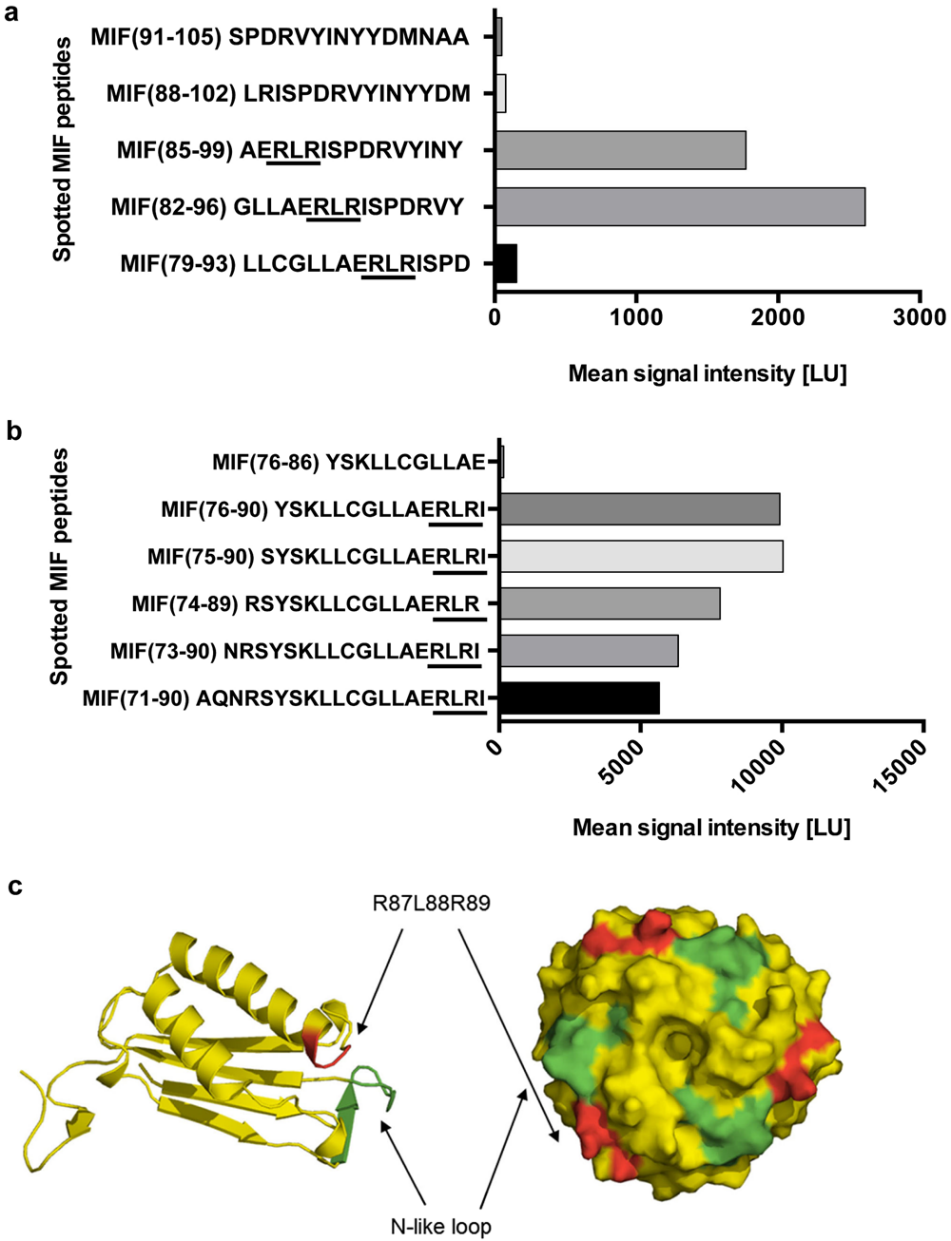
Identification of the RLR sequence as a potential MIF binding region to the N-terminal peptide of CXCR4. (**a**,**b**) The peptide spot microarray method suggests that the RLR tripeptide at sequence position 87–89 may contribute to MIF/CXCR4 binding. A peptide spot array containing 15-mer spotted MIF peptides positionally shifted by three amino acids were probed with biotin-CXCR4(1–27). Graphs are plots of spotted MIF peptides over the intensity of the binding signal to biotin-CXCR4(1–27) as read-out by streptavidin Cy5.5 fluorescence. (**a**) Of five positionally shifted 15-mer peptides of the region 79–105 only peptides containing RLR interact with CXCR4(1–27). (**b**) Binding of RLR-containing MIF peptides is modulated by N-terminal extension, but residues N-terminal of RLR do not exhibit binding activity *per se*. (**c**) Structural model of MIF (as monomer and trimer) and position of the N-like loop (green) and the RLR sequence (red). Note: in the three-dimensional conformation of the monomer, RLR is located in the vicinity of the N-like loop of MIF. The trimeric structure shows that both the N-like loop and RLR are surface-exposed on the trimer (see also Fig. 7).

Peptide spot array analysis of the C-terminal region of the extended N-like loop of MIF using immobilized 15-mer human MIF peptides with each peptide positionally shifted by three residues, was probed for binding to biotinylated CXCR4 N-terminus (CXCR4(1–27)). Analysis revealed a marked signal for MIF peptides 82–96 and 85–99, while giving a small signal for peptide 79–93 and no signal for peptides 88–102 and 91–105 (Fig. 1a). Among the residues shared by the responsive peptides was the Arg-87-Leu-88-Arg-89 (RLR) sequence segment. Considering sequences comprising basic residues such as Arg or Lys have been found to contribute to the site 1 interaction surface between CXCL12 and CXCR4^39^, we hypothesized the RLR region may contribute to MIF/CXCR4 binding. To further test the potential relevance of this sequence, the influence of N-terminal or C-terminal neighboring amino acids, and peptide length, we also analyzed peptides 71–90, 73–90, 74–89, 75–90, 76–90. Peptide 76–86 was used as an RLR-void control. Overall, this analysis confirmed a role for the RLR sequence of MIF in CXCR4(1–27) binding. While the comparison of the tested peptides in Fig. 1a also implied that a net positive charge (+1 versus 0 or −1) may foster the interaction with CXCR4(1–27), the extended comparison of probed peptides in Fig. 1b suggested that signal strength also is modulated by N-terminal extension of the RLR sequence, while N-terminally extended peptides with a net positive charge of +2 showed higher signal intensities than the shorter peptides with a net charge of +3 (Fig. 1b). A more specific role for the RLR sequence also was confirmed by a control experiment using randomized - ‘scrambled’ – peptides. Binding of biotin-CXCR4(1–27) to peptide 75–90, i.e. the strongest interacting MIF peptide tested, was compared with the binding of five randomized - ‘scrambled’ – sequences of MIF peptide 75–90. Supplementary Fig. 1 shows that binding of the scrambled peptides to CXCR4(1–27) was markedly lower than binding of the RLR-containing wildtype sequence.

When we inspected the position of RLR in the three-dimensional structure of MIF, it appeared that residues 87–89 are positioned in the vicinity of the N-like loop of MIF. RLR is located at the C-terminal end of the second α-helix, with the two arginine residues of RLR being surface-exposed and the leucine making hydrophobic contacts (see ref.)^36^. Overall, this may lead to an expansion of the surface of the N-like loop, both in the monomeric and trimeric structures of MIF (Fig. 1c). Interestingly, CXCR4(1–27) comprises seven negatively charged residues (Glu-2, Asp-10, Glu-14, Glu-15, Asp-20, Asp-22, Glu-26) that could qualify for interactions with the positive charges within RLR of MIF. Therefore based on these initial data, we hypothesized that the RLR sequence stretch could contribute to the site 1 binding region between MIF and its chemokine receptor CXCR4.

**Figure 2:**
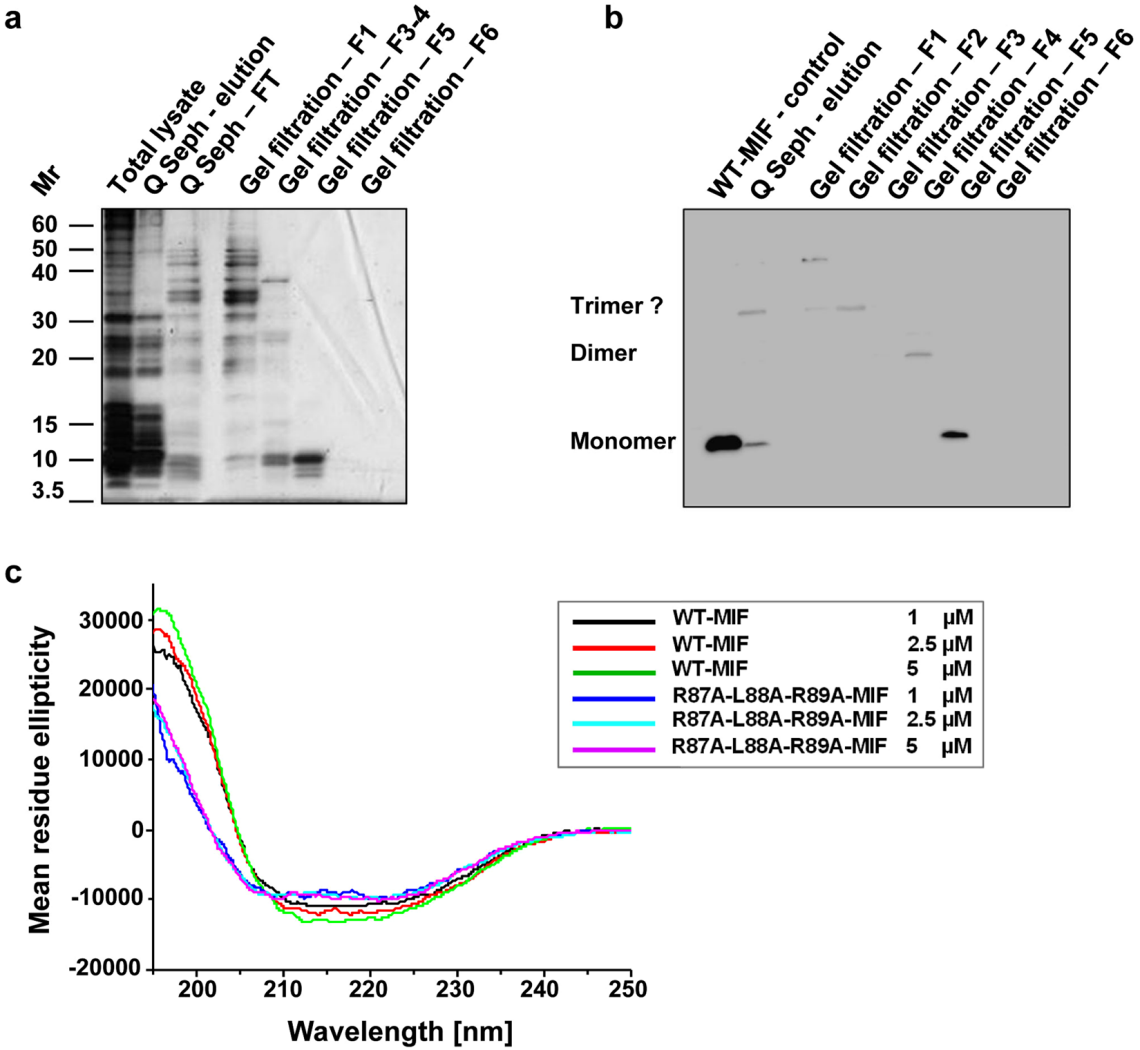
Expression, purification, and conformational integrity of R87A-L88A-R89A-MIF. (**a**) Recombinant expression and purification of R87A-L88A-R89A-MIF by anion exchange chromatography (Q Seph) and size exclusion chromatography (SEC, gel filtration) as analyzed by SDS-PAGE and silver staining. Total lysate, bacterial lysate after IPTG induction; Q Seph - elution, specific elution of protein from Q sepharose column by increasing salt gradient; Q Seph – FT, flow-through; gel filtration F1-F5, elution fractions 1–5 (see Supplementary Fig. 3). (**b**) Same as (**a**) but analysis by Western blot using a polyclonal anti-MIF antibody. (**c**) Circular dichroism (CD) spectropolarimetry shows that the folding and secondary structure profile of R87A-L88A-R89A-MIF is overall similar to that of WT-MIF. Spectra of R87A-L88A-R89A-MIF and WT-MIF at different concentrations are presented according to the indicated color code. Conformations in the CD spectra were measured as mean residue ellipticity versus the wavelength in the far-UV range.

A triple alanine mutant of MIF (R87A-L88A-R89A-MIF) was expressed in *E. coli* BL21/DE3. The expression efficiency of the mutant was lower than that of WT-MIF. The mutant was recovered from the cleared bacterial lysate and did not form inclusion bodies. It has a predicted lower isoelectric point than WT-MIF (pI (R87A-L88A-R89A-MIF) = 6.1) compared to 7.73 for WT-MIF and a predicted higher grand average of hydropathy (GRAVY) (0.090434 versus −0.001739; Supplementary Fig. 2). Accordingly, we chose a purification strategy that was different from that established for WT-MIF^40^. Whereas WT-MIF does not bind to an anion exchange column at pH 7.5^40^, R87A-L88A-R89A-MIF interacts with the anion exchange material under these conditions. The mutant protein was eluted from the anion exchange column via salt gradient and subjected to size exclusion chromatography (SEC) for further purification. The RLR mutant eluted at peaks with an approximate molecular size of 23 and 38 kDa, suggesting that it forms dimers and trimers under these conditions (Supplementary Fig. 3). Similar to WT-MIF^41,42^, these oligomers constituted the major peak(s) in the size exclusion chromatogram, but the mutant additionally also showed several high molecular weight peaks (52–124 kDa), likely representing high molecular weight oligomers or aggregates. Applying this procedure, the mutant protein was obtained in appreciable yield, purity, and free of endotoxin contamination, as confirmed by SDS-PAGE silver-staining and Western blot analysis of the obtained fractions (Fig. 2a,b and Supplementary Table 1).

Next, we performed circular dichroism (CD) spectropolarimetry to address the question whether the alanine substitutions at the RLR site affect the structural integrity of MIF. Far-ultraviolet (UV) spectra were recorded between 195 and 250 nm and the spectrum of the RLR mutant compared with that of WT-MIF. CD spectroscopy provides a good estimation of the secondary structural profile of a protein and records relative changes in average secondary structural content, e.g. when a mutated protein is compared to its wildtype counterpart. Figure 2c shows that the secondary structure profile of the RLR mutant is similar to that of WT-MIF. Significant changes were limited to the spectral region below 210 nm. Dose-dependent recordings at 1, 2.5, and 5 µM did not lead to substantial spectral changes, neither for WT-MIF nor for the triple alanine mutant, indicating that neither protein showed an aggregation-tendency in this concentration range. Moreover, the quantification of the secondary structural contents following deconvolution of the CD spectra applying Dichroweb^43,44^ indicated that approximately 80% of the average ordered secondary structural content remained unaffected by the alanine substitutions (Supplementary Table 2). The deconvolution of the CD spectra also indicated that the typical α/β-structure of WT-MIF^36,40^ was preserved in the mutant, although the α-helix content, and to a lower extent, the β-strand percentage, in the mutant protein were found to be reduced by 12% and 7%, respectively, compared to the WT-MIF protein (Supplementary Table 2). In turn, random coil content was increased in the mutant by approximately 20%. Overall, the biochemical and biophysical data indicated that the alanine substitutions did not substantively interfere with the overall structural integrity of the MIF protein, although smaller changes of the average secondary structural content were observed and slight alterations on tertiary structure level cannot be excluded.

### The RLR residues contribute to MIF/CXCR4 binding and the CXCR4-mediated cellular signaling activity of MIF

We previously determined the binding interaction between Alexa-488-labeled WT-MIF and CXCR4(1–27) by fluorescence spectroscopic titration. The K_d_ was estimated to be in the range of 10 µM^35^. Here, we performed fluorescence titrations with different concentrations of CXCR4(1–27) and Alexa-488-labeled R87A-L88A-R89A-MIF. The fluorescence emission of Alexa-488-labeled R87A-L88A-R89A-MIF at 522 nm did not exhibit any dose-dependent changes up to a 763-fold molar excess of CXCR4(1–27) (Supplementary Fig. 4). This suggested that R87A-L88A-R89A-MIF does not bind to CXCR(1–27) and supports the notion that the RLR peptide contributes to MIF/CXCR4 binding.

**Figure 3:**
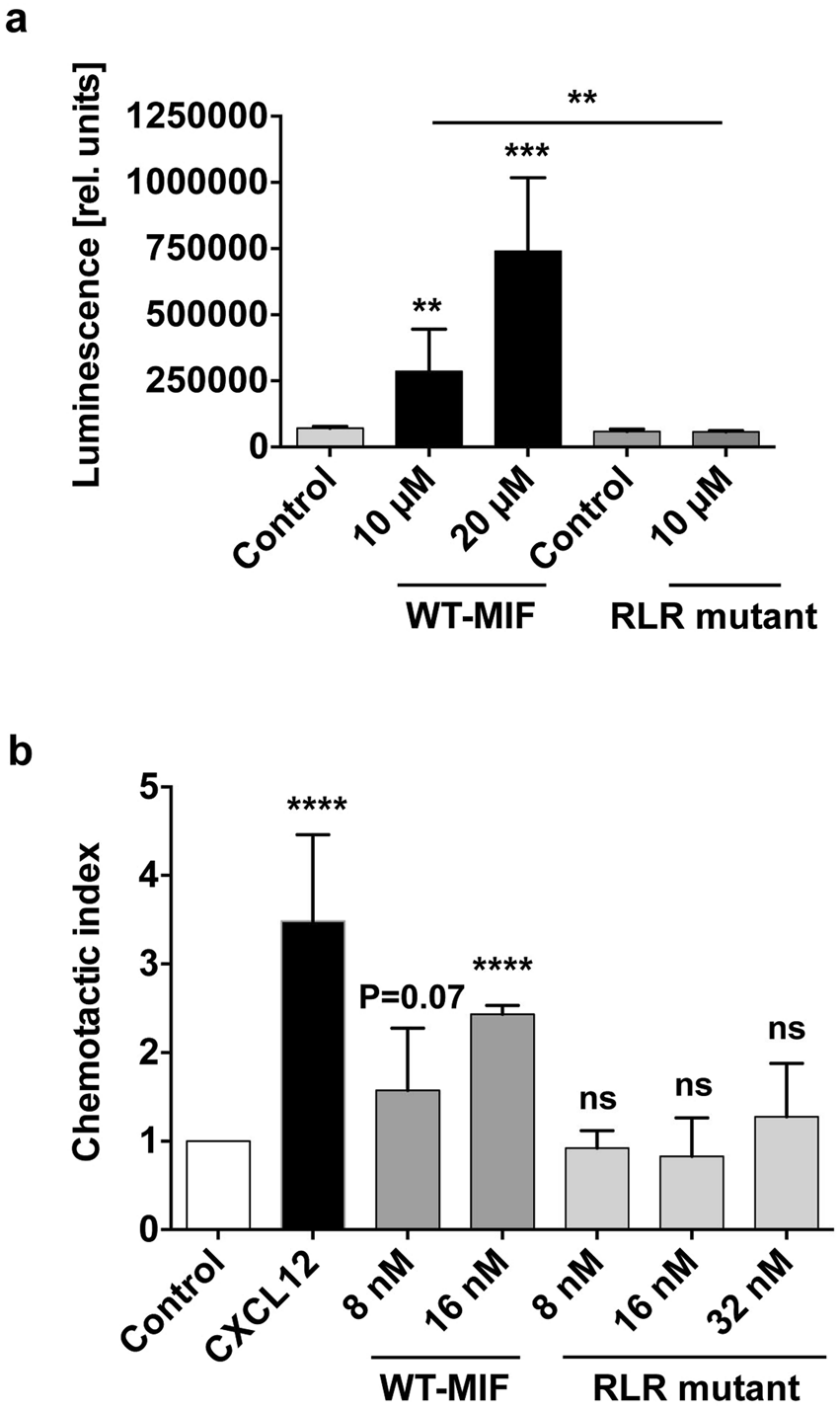
Mutation of the RLR residues markedly impairs CXCR4-dependent MIF cell signaling activities. (**a**) MIF signaling through CXCR4 in *S. cerevisiae* is abolished in R87A-L88A-R89A-MIF. CXCR4 replaces the Ste 2 receptor pheromone response pathway of *S. cerevisiae*. The pathway is further modified to enable activated human CXCR4 to elicit a robust signaling response that couples to the expression of different levels of lacZ gene dependent on the level of signaling, which is measured by enzymatic activity. Enzymatic activity is represented as relative luminescence. The concentrations of WT-MIF and RLR mutant are as indicated. Corresponding control buffers were used for WT-MIF (control 1) and the RLR mutant (control 2). Values are means ± SD of 3–9 replicates representing three-to-four experiments. (**b**) Impaired chemotactic effect of R87A-L88A-R89A-MIF in CXCR4-dependent lymphocyte migration. Chemotactic migration (represented as chemotactic index) of JVM-3 lymphocytes towards R87A-L88A-R89A-MIF is compared with that elicited by WT-MIF at the indicated concentrations and CXCL12 (8 nM). PBS buffer was used as negative control to normalize for spontaneous random migration (control). The bar graph shows means ± SD of 3–5 experiments. **p < 0.01; ***p < 0.005; ****p < 0.001.

To address the contribution of the RLR motif to MIF/CXCR4-specific cell signaling responses, we employed a genetically modified strain of *Saccharomyces cerevisiae* that replaces the yeast ste2 GPCR with human CXCR4 as previously reported^35^. This cell system eliminates complications from mammalian cells that usually express more than one or all MIF cell surface receptors and is based on agonist-mediated activation of CXCR4 leading to a signaling cascade that results in β-galactosidase expression from the Fus1-lacZ reporter plasmid. We recently demonstrated that MIF activates CXCR4 signaling in this system. MIF agonism is similar but not identical to that of CXC12, exhibiting partial allosteric agonism in comparison with CXCL12^35^. Recombinant WT-MIF but not a control buffer triggered a marked CXCR4 response as previously reported^35^ (Fig. 3a). In contrast, R87A-L88A-R89A-MIF failed to activate CXCR4 (Fig. 3a), confirming that the RLR sequence is involved in MIF/CXCR4 binding, and suggesting that it is necessary for MIF/CXCR4-mediated cell signaling.

Although the yeast cell system elegantly bypasses the problem of multiple MIF receptor expression in mammalian cells, it is somewhat artificial in that yeast features an outer cell wall in addition to the plasma membrane, necessitating the use of micromolar concentrations of agonists due to impaired access to CXCR4 in the membrane^35,45^. We thus next wished to confirm and extend these findings in a cell system with an even higher physiological and pathophysiological relevance. One major function of the MIF/CXCR4 axis is to support the chemotactic recruitment of T and B lymphocytes^30,32,46,47^. Here, we employed the human B cell line JVM-3, which expresses significant levels of the MIF receptors CXCR4 (Supplementary Fig. 5) and CD74, but not CXCR2 or CXCR7 (T. Thavayogarajah, D. Sinitski, and J. Bernhagen, unpublished observation) and is an established cell model to monitor chemokine-mediated chemotactic migration responses^48^. Figure 3b demonstrates that CXCL12 elicited a marked JVM-3 chemotaxis response (CTX_CXCL12_ = 3.5 ± 0.4, p < 0.0001). The chemotactic effect of WT-MIF was lower than that of CXCL12 as previously seen, followed a concentration-dependence, and was similar to previously observed chemotactic effects of MIF on mouse B cells^46^ (8 nM: CTX_MIF_ = 1.58 ± 0.4, p = 0.07; 16 nM: CTX_MIF_ = 2.43 ± 0.06, p < 0.0001). The RLR mutant did not exhibit a significant chemotactic activity on JVM-3 B cells at any of the concentrations tested (8 nM, 16 nM, 32 nM; p = ns; Fig. 3b). This suggested that the RLR sequence segment significantly contributes to MIF/CXCR4-mediated chemotactic cell migration responses.

### Direct involvement of the RLR sequence in MIF/CXCR4 interactions

**Figure 4:**
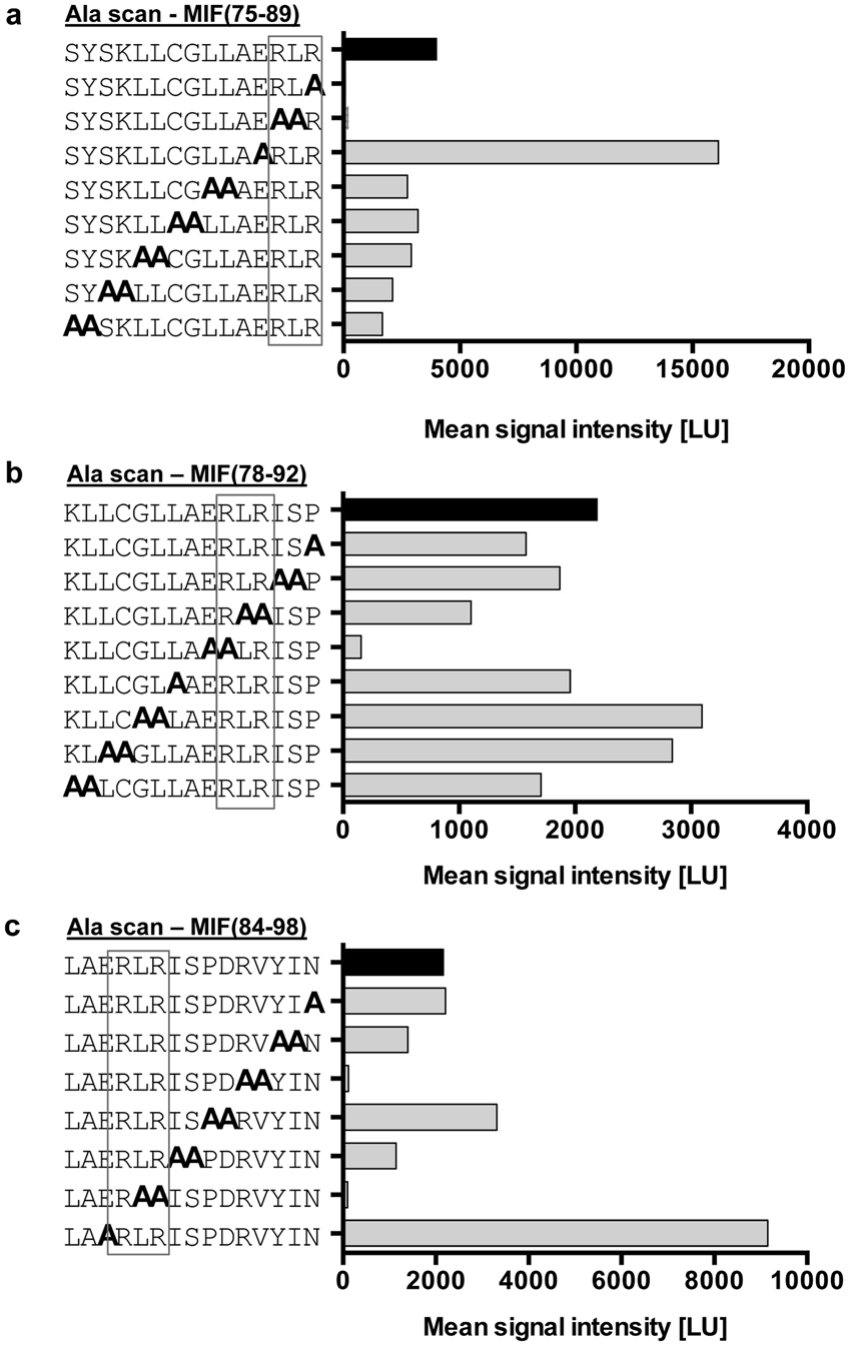
Alanine scanning of RLR-containing MIF peptides reveals role for RLR in binding the CXCR4 N-terminal peptide 1–27. A peptide spot array containing spotted 15-mer MIF peptides (**a**) 75–89, (**b**) 78–92, and (**c**) 84–98 was subjected to alanine scanning and probed with biotin-CXCR4(1–27). Sequential mono- or di-alanine substitutions were performed across the entire sequence of the peptides as indicated. Graphs are plots of spotted MIF/Ala scan peptides over the intensity of the binding signal to biotin-CXCR4(1–27) as read-out by streptavidin Cy5.5 fluorescence. Bars for wild-type peptides without alanine substitution are depicted in black; the position of the RLR sequence is framed.

To obtain evidence that the RLR residues are directly involved in the MIF/CXCR4 interface, we combined the peptide spot array technology of the C-terminal region of the extended N-like loop of MIF with an alanine scanning approach. 15-mer human MIF peptides 75–89, 78–92, and 84–98, containing RLR in the C-terminal, middle or N-terminal part of the sequence, respectively, were synthesized as either wildtype sequence or as one of nine different alanine scan variants, with one or two alanine substitutions introduced across the entire sequence length (Fig. 4). Immobilized alanine scan variants were then probed for binding to biotinylated CXCR4(1–27). As expected from the peptide array analysis of RLR-containing MIF 15-mer peptides (Fig. 1**)**, wild-type peptides 75–89, 78–92, and 84–98 bound CXCR4(1–27) with signal intensities between 2000 and 4000 LU. Overall, alanine scanning supported the notion that only alanine substitutions of the RLR region itself led to marked reductions in CXCR4(1–27) binding activity (Fig. 4a–c). This is most apparent in peptides 75–89 and 78–92. An exception is the alanine substitution of residues 94 and 95 in peptide 84–98. Interestingly, residue 94 in WT-MIF is an arginine, insinuating that another positively charged amino acid in the vicinity of RLR may further contribute to MIF/CXCR4 binding. One other intriguing observation was made in the alanine scanning experiment. The substitution of the glutamic acid (residue 86) immediately preceding RLR in peptides 75–89 and 84–98 led to a pronounced increase in the binding signal (Fig. 4a,c). The substitution of glutamic acid by alanine at this position eliminates a negative charge in the vicinity of RLR, supporting the conclusion that a net positive charge within RLR or in its immediate vicinity is important for CXCR4(1–27) binding. Altogether, these data show that only alanine substitutions within or in the vicinity of RLR lead to alterations in CXCR4(1–27) binding activity, suggesting a direct role for this MIF region in the MIF/CXCR4 binding interface.

**Figure 5:**
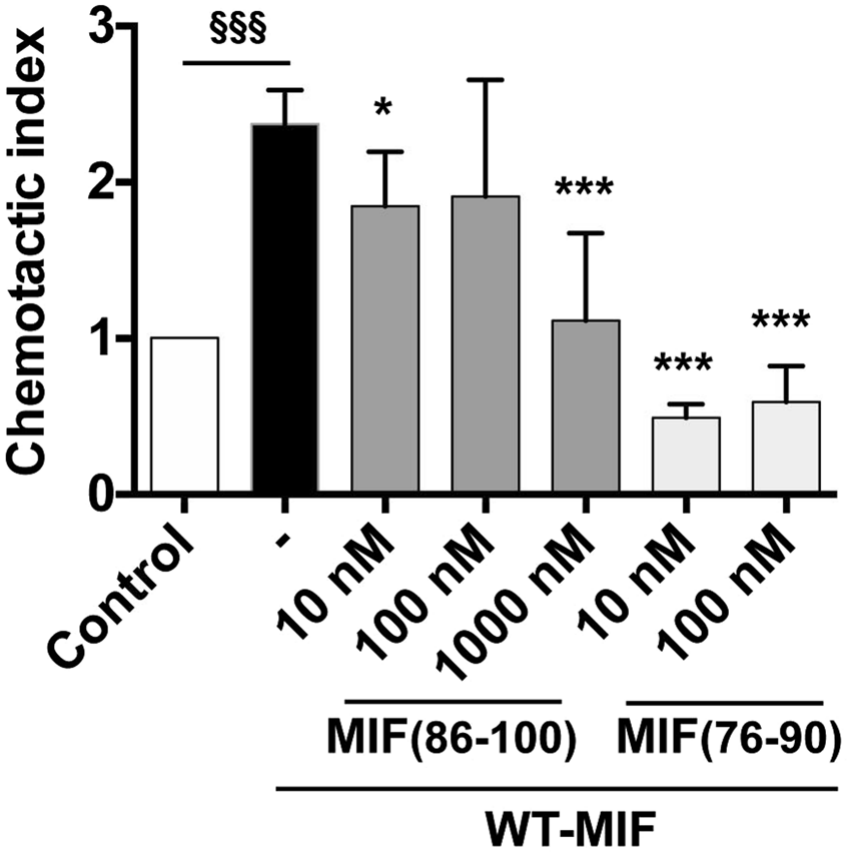
RLR-containing 15-mer MIF peptides block MIF/CXCR4-dependent JVM-3 lymphocyte chemotaxis. Chemotactic migration (represented as chemotactic index) of JVM-3 lymphocytes towards WT-MIF (16 nM) in the presence versus absence of different concentrations of RLR-containing peptides MIF(76–90) or YSKLLCGLLAERLRI and MIF(86–100) or ERLRISPDRVYINYY as indicated. PBS buffer was used as negative control to normalize for spontaneous random migration (control). The bar graph shows means ± SD of 3–12 replicates and represent 2–3 experiments. Statistical comparisons were done between the MIF and MIF + peptide data sets (*p < 0.05; ***p < 0.005) and between control and MIF (^§§§^p < 0.005).

To confirm this notion, we asked whether the RLR-spanning peptides MIF(86–100 or E**RLR**ISPDRVYINYY) and MIF(76–90 or YSKLLCGLLAE**RLR**I), i.e. peptide sequences with strong binding signals in the peptide array experiments and a representing more N-terminal and C-terminal RLR positions, respectively, are able to interfere with MIF-mediated JVM3 B cell chemotaxis. The competition experiments adding different concentrations of the RLR-spanning peptides together with 16 nM of full-length WT-MIF into the chemotaxis chambers showed that both peptides competed with WT-MIF, leading to a reduction/ablation of the CXCR4-dependent chemotactic activity of MIF (Fig. 5). Peptide MIF(86–100) showed a concentration-dependent competition behavior with a trend towards inhibition seen at concentrations of 10 and 100 nM, and a significant and complete blockade of MIF activity at a concentration of 1 µM. Peptide MIF(76–90) was even more potent and ablated the CXCR4-dependent chemotactic activity of MIF at concentrations of 10 and 100 nM. Together, the peptide competition experiment corroborated the notion that the RLR region directly contributes to functional MIF/CXCR4 interactions.

**Figure 6:**
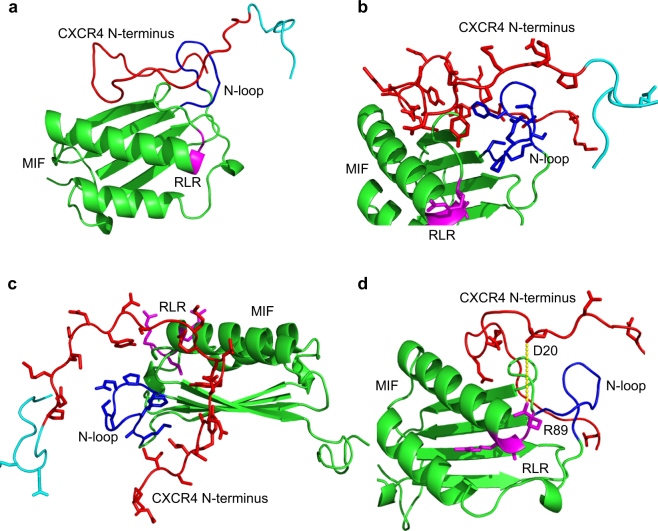
Structure model of the complex between human MIF and the N-terminus of CXCR4 as determined by molecular docking. Ribbon structures of the complex between full-length human MIF and human CXCR4(1–38) as determined by molecular docking are shown in different orientations with and without the representation of side chains. **(a**) Ribbon structure of the complex between MIF and CXCR4(1–38) without depicting any side chains. The following color code was used: MIF structure (green), CXCR4 N-terminus (red), RLR sequence of MIF (magenta), N-like loop of MIF (blue), residues 28–38 of CXCR4 (turquoise). (**b**) Same as (**a**) in slightly rotated orientation with relevant side chains of RLR, N-like loop, and CXCR4 N-terminus visualized. For clarity reasons, these residues are not labeled. (**c**) Side view of the structure in (**b**). (**d**) Same as (**a**) but with focus on the negatively charged residues in CXCR4(1–27), i.e. Glu-2, Asp-10, Glu-14, Glu-15, Asp-20, Asp-22, Glu-26, and the two positively charged arginine residues in RLR. A potential interaction between Arg-89 (R89) in RLR and Asp-20 (D20) of the CXCR4 N-terminus is indicated by a yellow dotted line.

Finally, we tested this structural concept by molecular docking simulations. The N-terminal 22 or 26 amino acids of CXCR4, respectively, did not have interpretable density in the available CXCR4 X-ray structures^5,6^. However, a recent nuclear magnetic resonance (NMR)-based structure of the complex between CXCR4(1–38) and a CXCL12 monomer provides structural information about the amino terminus of human CXCR4^49^. We subjected this structure of the CXCR4 N-terminus (2n55 CXCR4 Nterm.pdb) and the structure of human MIF (hMIF 3djhmonomer.pdb) to the PATCHDOCK molecular docking algorithm that is based on shape complementarity principles. Both the restricted analysis, confined to residues 1–27 of the N-terminus, and the unrestricted approach, covering all 38 amino acids of the N-terminus, provided similar results in the PATCHDOCK calculation (data not shown) and were submitted to FIREDOCK for refinement and rescoring. For the highest ranked docking solution, a global energy of −43.76 kcal/mole was obtained for the MIF/CXCR4(1–38) complex. The following 10 lower ranked solutions also had global energies >30 kcal/mole. Similar results were obtained for the complex with CXCR4(1–27). Figure 6 illustrates the structures obtained for the highest ranked docking solution of the complex between human MIF and the CXCR4 N-terminal. The molecular docking result confirms the notion that the N-terminus of CXCR4 could engage in multiple direct interactions with both the N-like loop and the RLR tripeptide area (Fig. 6a–c). Moreover, GPCRs are known to be dynamic and the N-terminal of CXCR4 is conformationally flexible^5,6,49^, facilitating dynamic ligand contacts. A potential charge interaction between Arg-89 of the MIF RLR sequence and Asp-20 of the CXCR4 N-terminal is indicated (Fig. 6d).

**Figure 7:**
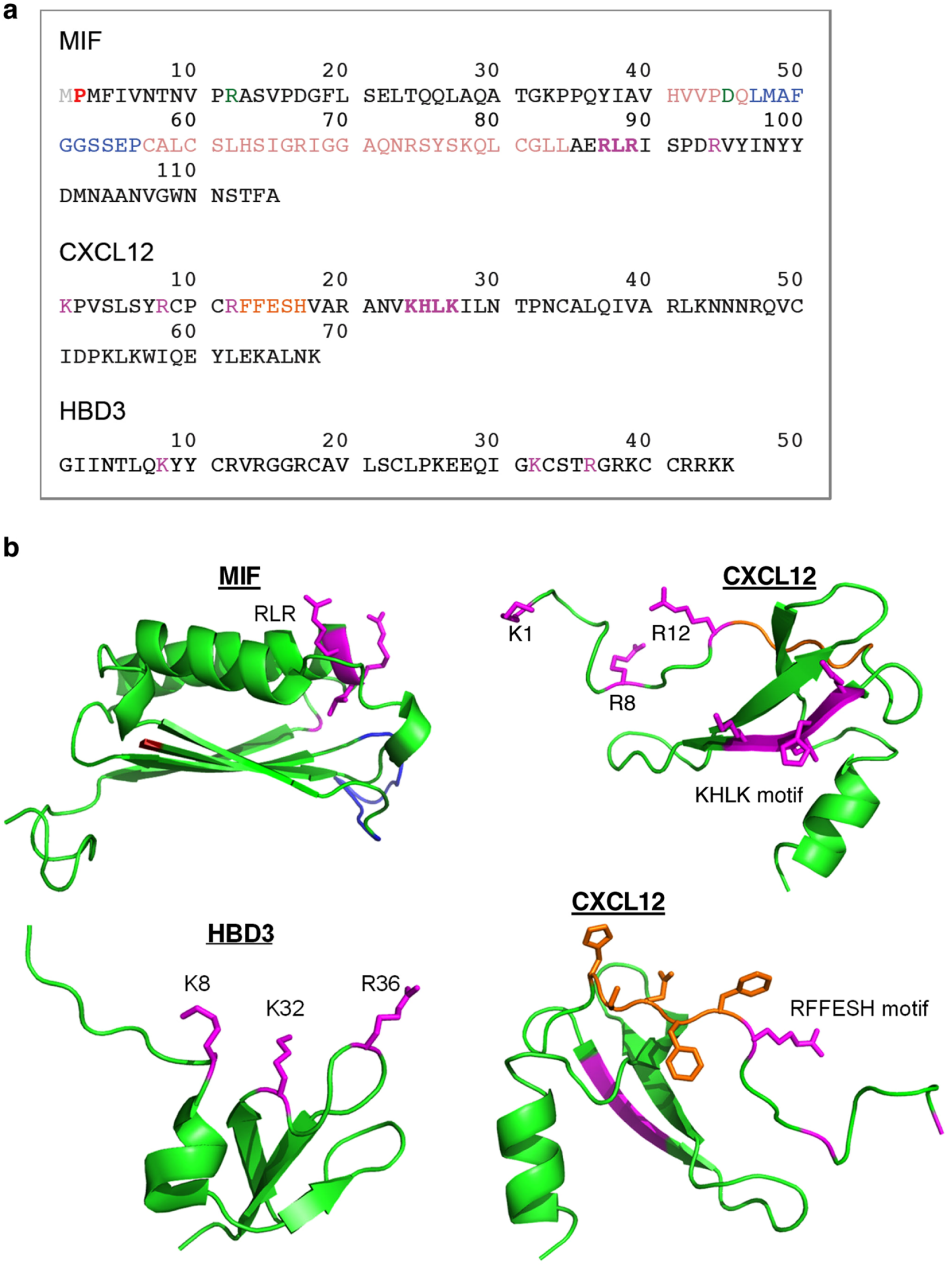
Structure comparison of MIF, CXCL12, and human β-defensin-3. **(a**) Comparison of the amino acid sequences, highlighting residues/motifs that have been implicated in binding to CXCR4. MIF: RLR (magenta, bold), Arg-94 (R94; magenta), N-like loop (suggested to contribute to site 1 binding^35^; blue), Pro-2 (suggested to contribute to site 1 binding^35^; red), extended N-like loop (suggested to contribute to site 1 binding^35^; beige), pseudo-ELR motif (discontinuous, contributes to MIF/CXCR2 interface; green), Met-1 (cleaved upon expression; grey). CXCL12: KHLK motif (magenta, bold), Lys-1/Arg-8/Arg-12 charge cluster (magenta), RFFESH motif (orange). HBD3: Lys-8/Lys-32/Arg-36 charge cluster (magenta). (**b**) Comparison of the three-dimensional structures. Ribbon structures of the respective monomers are shown, with the backbones depicted in green. The corresponding color code and the captions for the residues/motifs that have been implicated in CXCR4 binding are as in (**a**); in the MIF structure, the ‘extension’ of the N-like loop (beige color in Fig. 7a) is not colored and the processed N-terminal methionine is not shown for clarity reasons. CXCL12 is depicted in two views (i) to highlight the KHLK motif and the charge cluster (top right) and (ii) the RFFESH motif (bottom right). Protein structures were produced/visualized with PyMOL.

**Figure 8:**
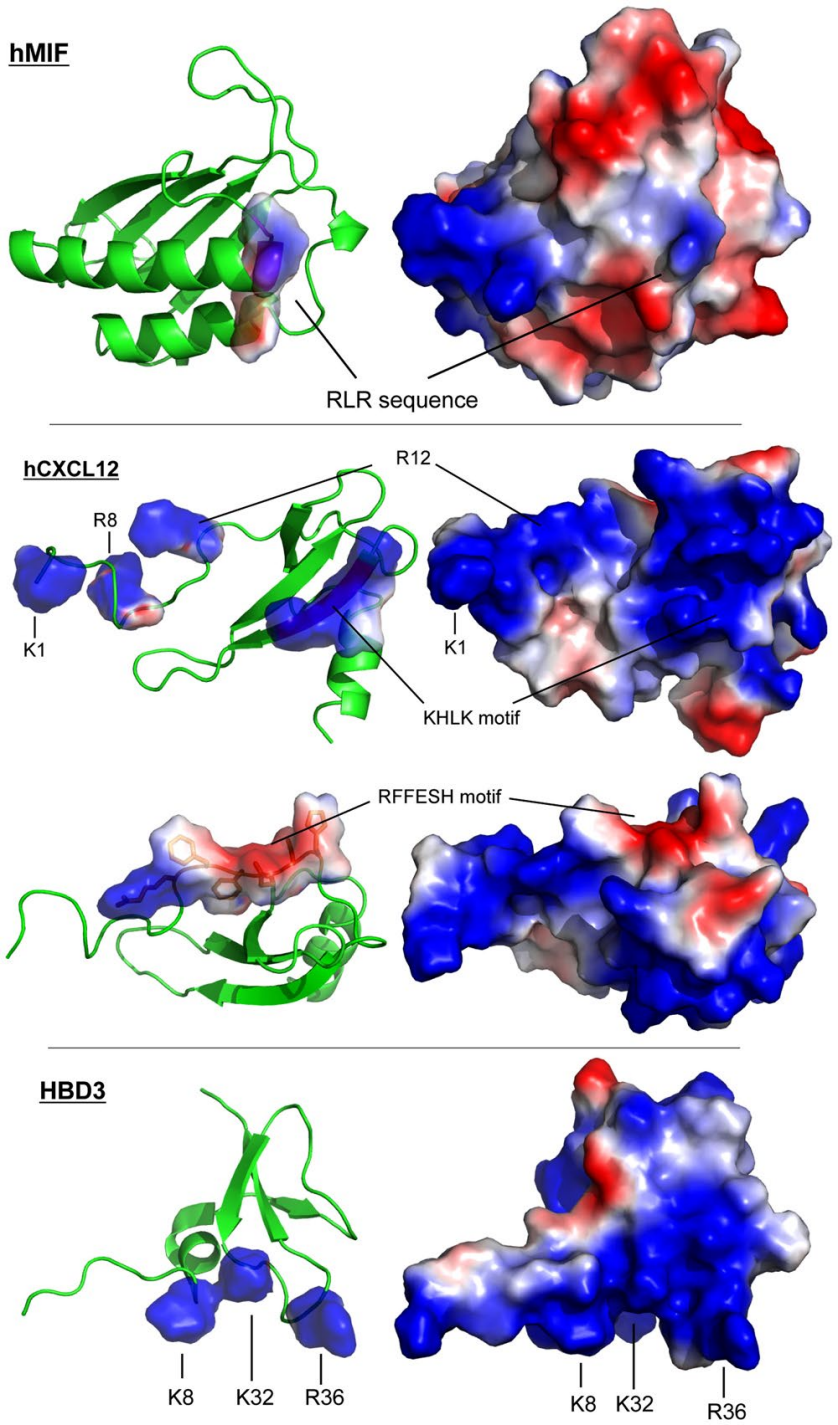
Comparison of the surface charges of MIF, CXCL12, and human β-defensin-3. Comparison of the three-dimensional structures of MIF, CXCL12, and human β-defensin-3 (HBD3) focusing on the surface charges. The relevant positively charged residues, the RLR, and the KHLK sequence, as well as the RFFESH motif are indicated as shown. Blue, positively charged residues arginine, lysine, or histidine; red, negatively charged residues aspartate and glutamate. Protein structures were produced/visualized with PyMOL.

Overall, these data suggest that the RLR region directly contributes to the MIF/CXCR4 binding interface to control CXCR4-mediated cellular effects of MIF. Charge interactions could play an important role in stabilizing the binding interface. In fact, the interaction between CXCR4 and its known ligands CXCL12 and human β-defensin-3 (HBD3) involves charge clusters^39,50^. We have compared the sequence and structure of MIF with that of the CXCR4 ligands CXCL12 and HDB3 and analyzed the positions of residues, motifs, and charge clusters that have been implicated in the interaction with CXCR4 (Figs 7 and 8, and Supplementary Fig. 6). Figure 7a and Supplementary Fig. 6 highlight the residues and motifs in MIF that have previously been implicated in site 1 and 2 interactions between MIF and CXCR4 and compare them to the presumed corresponding residues in CXCL12 and HBD3. Of note, the MIF RLR sequence could represent a positive charge cluster similar to those represented by the K1-R8-R12 residues or the KHLK motif in CXCL12 as well as the K8-K32-R36 cluster in HBD3 (Fig. 7)^39,50^. This notion is supported when comparing the surface charges in these regions between MIF, CXCL12, and HBD3 (Fig. 8).

## Discussion

MIF is a pivotal inflammatory cytokine^18,20,25^ and prototypical atypical chemokine (ACK) that exhibits key ACK features^24,25,30^. This study identifies residues in MIF, namely the Arg-Leu-Arg (RLR) tripeptide region, that contribute to site 1 binding of the MIF/CXCR4 interface to control CXCR4-mediated cellular effects of MIF. With an N-like loop and the Pro-2 residue previously identified to support MIF/CXCR4 binding^35^, the current study completes the picture as to how an ACK such as MIF engages a classical chemokine receptor (CKR) such as CXCR4 and will help to mechanistically understand differences versus similarities between MIF-mediated CXCR4 signaling and that of the cognate ligand.

Chemokine receptors control numerous cellular pathways and orchestrate leukocyte trafficking under physiological and pathophysiological conditions. Hence, CKs and their receptors have been implicated as pivotal mediators in numerous diseases, e.g. inflammatory and cardiovascular diseases, autoimmune conditions, or cancer. As prototypical GPCRs of the Gαi subtype, CKRs are druggable and numerous small molecule and peptide-based inhibitors are being pursued as potential therapeutic candidates, but considerable challenges remain^51,52^. In particular, it has been difficult to devise CKR-specific approaches. The specificity problem occurs partly because the same receptor is expressed by different cell types but is activated by different chemokines expressed in various tissues and can potentially lead to adverse effects^4^. Accordingly, chemokine signaling mechanisms underlying ligand, receptor, and tissue bias are extensively studied.

The ACK activities of MIF contribute to its key role in inflammatory and cardiovascular diseases and cancer. For example, the MIF/CXCR2 axis controls atherogenic monocyte recruitment^30^ and natural killer T (NKT) cell migration in inflammatory skin conditions^53^. CXCR4-mediated MIF cell signaling activities encompass atherogenic lymphocyte recruitment^30,32,46^, ischemia-triggered endothelial progenitor cell migration^54,55^, eosinophil inflammation^56^, promotion of inflammatory platelet survival^57^, or colon cancer cell metastasis^58^. It would thus be desirable to therapeutically interfere with these pathophysiological activities. However, there are multiple homeostatic and ‘beneficial’ functions mediated by CXCR4 following activation with its *bona fide* ligand CXCL12 such as cell homing, cardiac development, or neutrophil egress^24,59,60^. Also, CXCR4 can form receptor complexes with CD74^30,61^, and MIF/CD74 signaling has important cardioprotective activities in myocardial ischemia/reperfusion injury^62,63^. In atherosclerosis, recent cell-specific knockouts of CXCR4 as well as the study of neutrophil-mediated atherogenic effects has established that the CXCR4/CXCL12 axis has cell-dependent protective or exacerbating effects^64–66^.

Thus, to interfere with disease-promoting activities of the MIF/CXCR4 axis, specifically tailored targeting strategies need to be established that block MIF- but not CXCL12-mediated CXCR4 functions. This in turn requires a detailed structural understanding of the MIF/CXCR4 binding interface. We recently demonstrated that the unique N-terminal Pro-2 residue of MIF and its N-like loop region that MIF shares with classical chemokines are important determinants of the MIF/CXCR4 interface and that MIF activates CXCR4 by partial allosteric agonism compared with CXCL12^35^. However, these structural features can only partially explain the high binding affinity between MIF and CXCR4, and the various functional differences to CXCL12/CXCR4^24,30^. Although the reactivity of Pro-2 is unique and the cavity that contains it is an excellent druggable site^67^, Pro-2 is involved in MIF/CD74 binding as mutations of Pro-2 impair MIF signaling responses^68^. Inhibition at this site could be problematic in cardiovascular diseases, as MIF/CD74 signaling conveys cardioprotection in early ischemia/reperfusion injury in the heart via activation of the tissue-protective AMP kinase pathway^62,63^. MIF-targeting strategies should therefore ideally obviate Pro-2 and nearby residues as well as MIF region 79–85, both of which have been implicated in mediating CD74 activation^38,68^.

In the current study, we have addressed these requirements by further characterizing the MIF/CXCR4 interface. We have identified an important additional structural element that contributes to the MIF/CXCR4 interface and is required for critical CXCR4-mediated MIF functions. Using biochemical/biophysical techniques and structure-activity studies in conjunction with peptide array technology, CXCR4-controlled cell function analysis, alanine scanning, and molecular docking, we show that an RLR tripeptide located at position 87–89 of the MIF sequence is positioned in close proximity to the N-like loop of MIF, contributes to the MIF/CXCR4 binding interface, and is functionally involved in CXCR4-mediated MIF activities. The peptide array and docking experiments suggest an important role for charge interactions by RLR arginine residues, in line with the view that electrostatic interactions are important in protein-protein interactions in general and in CXCR4/ligand interactions in particular.

Initial evidence for a role of RLR was obtained by a peptide spot array experiment, when we tested the binding of numerous RLR-spanning 15-mer peptides to the N-terminal sequence of CXCR4 using biotin-CXCR4(1–27). Although the N-terminal sequence of CXCR4 is not ‘visible’ in the available X-ray crystallographic structures of CXCR4, it has also been implicated in interactions with the cognate ligand CXCL12 by structure-activity studies and a recent NMR spectroscopy structure of a complex between CXCL12 and the N-terminal peptide 1–38 of CXCR4 together with NMR/X-ray structure hybrid modeling suggested a distinct role for the receptor N-terminus in chemokine recognition and receptor activation^5,6,49^. Of note, the N-terminal region of CXCR4 had previously been implicated in MIF/CXCR4 binding and peptide CXCR4(1–27) was found to bind to WT-MIF with a K_d_ of approximately 10 µM^35^.

A fluorescence titration-based binding experiment in our current study showed that increasing concentrations of CXCR4(1–27) did not evoke conformational changes of a fluorescently labeled MIF mutant in which all three residues of the RLR sequence were substituted to alanine. Thus, we conclude that the triple mutant R87A-L88A-R89A-MIF does not bind to the CXCR4 N-terminus. This confirms the peptide array observations and suggests that the RLR sequence also is important for CXCR4(1–27) binding in the context of the three-dimensional –folded- MIF structure.

Importantly, the site-specific RLR mutant had a fully ablated CXCR4 signaling response in *S. cerevisiae* transformants specifically expressing CXCR4 and a reduced CXCR4-dependent lymphocyte migration response when compared with RLR-containing WT-MIF. The CXCR4-dependent yeast-signaling assay is a powerful functional tool^37^, as it represents a signaling-competent cell system that does not express any of the other MIF receptors. Accordingly, a signaling response can be directly linked to interactions between MIF and CXCR4. The MIF R87A-L88A-R89A triple mutant elicited a reduced cell migratory response in CXCR4-expressing JVM-3 lymphocytes compared to WT-MIF, but contrary to CXCR4-mediated signaling in the *S. cerevisiae* system the response was not completely abolished. While the mutant failed to show any chemotactic activity at the optimal concentration of WT-MIF (16 nM), a slight increase in activity was noted at 32 nM, although this effect did not reach statistical significance. The yeast only signals through a modified G-protein to induce expression of β-galactosidase, whereas JVM-3 cells signal through both G-proteins and β-arrestin. β-arrestin is known to have roles in cellular chemotaxis^69^. Another explanation for the subtle differences in WT-MIF versus triple mutant behavior between the yeast and JVM-3 chemotaxis cell systems could be that JVM-3 cells also express the MIF receptor CD74 (unpublished observations). Overall, ablation or reduction of MIF-mediated CXCR4-dependent cell responses by R87A-L88A-R89A-MIF in the yeast and JVM-3 lymphocyte assays suggests that the RLR sequence is critically involved in the MIF/CXCR4 interface.

The RLR mutant bioactivity data together with our structure-activity studies point toward a role for RLR in CXCR4 binding and CXCR4-mediated signaling. In fact, RLR may represent a novel hot segment in the binding interface^70^, but our study also has some limitations. The peptide spot array method is based on interactions of surface-immobilized 10–20-mer peptides with their binding partner. These peptides can only represent regions that are linear in nature with many different conformations. In our experiments, the RLR-containing peptides or their mutant counterparts were probed with biotinylated CXCR4(1–27). There are 7 acidic residues (Asp or Glu, including a di-Glu repeat) within the CXCR4 sequence 1–27 that could interact with the RLR motif by electrostatic/polar interactions. A role for electrostatic interactions between CXCR4(1–27) and MIF-RLR is further supported by our alanine scanning and the molecular docking results.

Alanine scanning shows that substitution of Glu-86, the acidic glutamic acid residue immediately preceding RLR, by alanine, causes a marked increase in the interaction signal between biotin-CXCR4(1–27) and the respective RLR-containing peptide. Although our alanine scanning and molecular docking data did not lend fully unanimous support to the notion that positive charges in or around the RLR sequence are the decisive factor of the binding force at the MIF/CXCR4-N-terminus interface, the E86A mutation was noticeable in that it led to an enormous increase in binding signal between MIF-RLR-containing peptide and biotin-CXCR4(1–27) in two independent MIF peptides (peptides 75–89 and 84–98), increasing the net positive charge in this region by +1. In the same vein, substitution of Arg-94 by alanine in peptide 84–98 led to a marked reduction in binding signal. The molecular docking approach between MIF and the CXCR4-N-terminus and the performed sequence and three-dimensional structure comparisons between MIF and CXCL12 as well as HBD3 confirmed the conclusion that positive charge interactions critically contribute to the site 1 interface between MIF and CXCR4. Arg-89 of MIF may engage in a binding interaction with Asp-20 of CXCR4 and numerous similarities were noted between the relevant positive charge clusters in CXCL12 (i.e. the KHLK motif) or HBD3 and the RLR area of MIF with its excess of net positive charges.

The alanine mutations of RLR do not interfere with the overall structural integrity of protein. To this end, CD spectroscopy of R87A-L88A-R89A-MIF confirmed the overall structural integrity of the folded mutant protein, although a 20% decrease in ordered secondary structural content was observed that was paralleled by a corresponding increase in unordered elements. However, CD can only determine average changes across all secondary structure elements of a protein and it is unknown whether e.g. the reduction in α-helix content affected the second α-helix of MIF, at the far C-terminal end of which RLR is located. Moreover, some of the available structures of human MIF place the RLR segment C-terminal of the α-helix^36^. Thus, RLR might reside in a less structured part of the MIF protein. Furthermore, although MIF has been suggested to interact with its receptor CD74 as a trimer^71^, it is currently unknown which oligomeric form binds to CXCR4. Monomeric and dimeric species of MIF have been reported^42,72,73^ and a MIF monomer may have a higher structural flexibility than the more rigid trimeric architecture. Our molecular docking experiments were performed with monomeric MIF, yielding reasonable global binding energies regarding the interaction with the N-terminal of CXCR4 and suggesting that the MIF RLR site and/or the N-like loop could engage in numerous direct interactions with the N-terminal 27 residues of CXCR4. The experiments performed in this study cannot fully rule out the possibility that the mutation of RLR leads to slight changes in the secondary or tertiary conformation of MIF, but the data obtained strongly point toward the conclusion that the three residues play a strong role in the interaction with CXCR4. The future X-ray crystallographic elucidation of the three-dimensional structure of R87A-L88A-R89A-MIF and eventually a co-crystal structure between WT-MIF and CXCR4(1–27) or full-length CXCR4 will clarify the precise positioning of RLR at the MIF/CXCR4 interface.

All five randomized control peptides of MIF(75–90) showed an impaired binding signal to CXCR4(1–27), when compared to the wildtype sequence featuring a preserved RLR site. This experiment suggested that, in addition to the net positive charge in the RLR region, the RLR sequence itself is critical for CXCR4 binding. Comparing the MIF/CXCR4 interaction with those of the other known CXCR4 ligands confirms this notion and further supports the role of the positive charge cluster in MIF. CXCL12 is one of the most basic chemokines, with an overall charge of +8. The corresponding net charge of the extracellular regions of CXCR4 is −9. Interestingly, the post-translational sulfation of tyrosine residues Tyr-7, Tyr-12, and Tyr-21 in the N-terminus of CXCR4, introducing additional negative charges, has been suggested to further enhance CXCL12 binding^74,75^, but the influence of post-translational CXCR4 modifications on MIF effects have not yet been studied.

With respect to the MIF RLR sequence, there is an HLK sequence (residues 25–27) in CXCL12. The strong increase in signal intensity of the ALRL mutant peptide as compared to a MIF ERLR peptide might thus correspond to the CXCL12 KHLK motif. Although in the absence of a MIF-CXCR4, nor CXCL12-CXCR4, structure it is difficult to know how MIF is oriented with respect to CXCR4, given that the MIF Glu-86 and CXCL12 Lys-24 are in the same position of the RLR and HLK residues of both proteins, respectively, gives support that this region is important for binding to CXCR4. The available vMIP-II-CXCR4 structure^5^ only is of limited usefulness in this regard. From a structural point of view, the vMIP-II residues at the position of the CXCL12 residues KHLK have no charge and are on the β-strand nearest to receptor interactions. However, it is not known what the subtle rotations of the proteins CXCL12 and CXCR4 relative to the vMIP-II-CXCR4 structure are. And it is far harder to predict what the structure of the MIF–CXCR4 complex is. Similar considerations apply to the charge cluster of HBD3^50^.

It needs to be emphasized that the binding sequences and properties of MIF, CXCL12, and HBD3 do not need to be exactly the same for binding to occur. This notion is borne out by the sequence of vMIP-II bound to CXCR4^5^ versus the sequence of CXCL12^39^ as well as the comparison of the vMIP-II-CXCR4 and CXCL12-CXCR4(1–38) co-structures^5,49^. Moreover, GPCRs are known to be very dynamic are able to accommodate different types of structures.

In summary, identification of the RLR tripeptide of the atypical chemokine MIF provides important structural information to our understanding of the MIF/CXCR4 binding site and helps to further distinguish this interface from that between CXCR4 and its cognate ligand CXCL12. This should also aid in the design drug-targeting approaches that are specific to MIF while leaving homeostatic CXCR4/CXCL12 or tissue-protective MIF/CD74 responses unaffected.

## Methods

### Cell culture, endotoxin assay, and reagents

JVM-3 cells (ACC-18) are a chronic B cell leukemia cell line and were kindly provided by Prof. M. Hallek, University of Cologne Medical School, Cologne, Germany. JVM-3 were grown in RPMI media with 1% penicillin/streptomycin and 10% fetal bovine serum (Invitrogen-Thermo Fisher Scientific, Karlsruhe, Germany) essentially as described previously^48^. Miscellaneous cell culture reagents also were bought from Invitrogen-Thermo Fisher Scientific and from PAA (Pasching, Austria). LPS content of the purified WT-MIF and RLR mutant proteins was tested by limulus amoebocyte assay (LAL, Lonza, Cologne, Germany) following the manufacturer’s recommendation. All other reagents were obtained from Sigma, Merck, Roth, or Calbiochem, and were of the highest purity degree available.

### Peptide synthesis

All peptides were produced by Fmoc solid phase synthesis (SPPS) and purified by high-performance liquid chromatography (HPLC) essentially as described previously^76^. The quality was checked by mass spectrometry analysis. Biotinylated N-terminal peptide human CXCR4(1–27) was synthesized by Fmoc-SPPS and biotin introduced N-terminally following an amino-caproic acid (Aca) spacer. RLR-containing MIF peptides 76–90 (YSKLLCGLLAERLRI) and 86–100 (ERLRISPDRVYINYY) were custom-synthesized by Peptide Specialty Laboratories (PSL, Heidelberg, Germany).

### Recombinant proteins, cloning of R87A-L88A-R89A-MIF, and SDS-PAGE/Western blotting

SDF-1α/CXCL12 was purified as previously described^77^. Biologically active recombinant human MIF (rMIF) was expressed and purified essentially as described^40^.

The RLR triple alanine mutant of MIF (MIF(R87A-L88A-R89A-MIF)) was cloned into the pET11b vector by site-specific mutagenesis using Quikchange II (Agilent, Waldbronn, Germany) and the cDNA of wildtype (WT) human MIF as template^40^ and expressed in *E. coli* BL21/DE3. Mutagenic primers were first synthesized to create single mutations within the the wild-type human MIF insert^40^. Subsequent mutagenic reactions created double mutant and triple mutant plasmids. Plasmids were sequenced to verify the accuracy and location of the mutations within the vector.

The RLR mutant protein was expressed in *E. coli* B21/DE3 (Merck-Novagen, Darmstadt, Germany). Cultures of 250 mL were grown at 37 °C until an optical density of 0.6–0.8 was reached. A final concentration of 1 mM isopropyl 1-thio-ß-D-galactopyranoide (IPTG) was used to induce protein expression for an additional 3.5 h. Bacteria were then harvested in aliquots of 50 mL by centrifugation, and the cell pellets were frozen at −20 °C for later use. For protein purification, cells pellets were resuspended in 2 mL Tris-based saline (25 mM Tris, 10 mM NaCl, pH 7.5). The bacteria were lysed under 75 mPa using a French Press (Emulsi-Flex C5, Avestin, Germany). Cell debris was removed by centrifugation at 38000 g for 30 min. To further reduce debris, the supernatant, referred to as the raw protein extract of the bacterial lysate, was sterile-filtered through a 0.22 μm membrane filter. Recombinant RLR mutant protein was first purified using anion exchange chromatography (Q sepharose, GE Healthcare, Freiburg, Germany) using a fast protein liquid chromatography system (FPLC, ÄKTA Pure, GE Healthcare). The system was equilibrated with Tris-buffered saline, pH 7.5. The triple mutant protein was eluted using a buffer gradient (ending in 25 mM Tris, 1 M NaCl, pH 7.5). Mutant protein-containing fractions were pooled and stored on ice. Following Q sepharose, the protein was further purified by size exclusion chromatography (SEC, Superdex 75, GE Healthcare) in 20 mM sodium phosphate buffer, pH 7.2, i.e. buffer conditions compatible with MIF bioactivity. R87A-L88A-R89A-MIF was sterile-filtered and stored at 4 °C until further use. The RLR mutant protein was obtained free of endotoxin contamination (<20 pg LPS/µg protein), suitable for subsequent biochemical and cell-based tests. Fractions containing the RLR mutant protein were confirmed by SDS-PAGE and Western blotting.

SDS-PAGE was performed in 15% gels under reducing conditions. Gels were either silver-stained or processed for Western blotting. For silver staining, gels were fixed for 16 h in 50% methanol/10% acetic acid in addition to 10% fixation enhancer (161–0461, BioRad, Munich, Germany). For Western blotting, proteins were transferred to nitrocellulose membranes at 20 V for 90 min. Blots were blocked with 5% bovine serum albumin and stained for MIF or RLR mutant bands using our rabbit polyclonal anti-MIF antibody (Ka345)^23^. Both gels and blots were imaged with the LiCor Odyssey Fc system (LICOR Biotechnology GmbH, Bad Homburg, Germany).

### CXCR4-specific yeast-signaling assay

The *S. cerevisiae* strain (CY12946) expressing a functional CXCR4 has been previously described^37^. Upon CXCR4 activation, MAP kinase signaling transcribes and translates β-galactosidase (lacZ), which is quantified by an enzymatic assay. To study the CXCR4 signaling by extracellular WT-MIF or R87A-L88A-R89A-MIF, CY12946 strain was transformed with CXCR4 in Cp4181 and β-gal in Cp1584. The transformed cells were grown overnight in selective medium. The cells were diluted to 0.3–0.8 OD_600 nm_ and incubated with WT-MIF or R87A-L88A-R89A-MIF. WT-MIF was tested at a final concentration of 10 and 20 μM, showing dose-dependent activation of CXCR4 signaling. Due to solubility restrictions of the corresponding stock solution, the triple alanine mutant could only be tested at a concentration of 10 µM. The activation of CXCR4 was quantitated by β-galactosidase activity using Beta glo kit (Promega). The data shown is the mean ± SD of 3–9 replicates representing three-to-four experiments.

### Peptide array methodology

The peptide microarray method using glass slide technology has been described previously^35^. Briefly, following stepwise SPOT synthesis (Intavis MultiPep RSi/CelluSpot Array, Cologne, Germany, or JPT, Berlin, Germany), the peptides were dispensed on an activated glass surface using a droplet-depositing system. Target peptides were immobilized chemo-selectively and purified by reaction of the peptides with the modified glass surface resulting in the formation of a covalent bond, which allowed the removal of all truncated and acetylated sequences by subsequent washing steps. After all peptides were arrayed on the glass surface, active residues were passivated. Analysis of interactions was performed using a microarray processing station (Intavis Slide Spotting Robot). The microarrays were incubated with biotinylated CXCR4(1–27) peptide. For determination of false-positives, one microarray was incubated with fluorescently-labeled streptavidin only. After incubation with 200 µl of biotinylated CXCR4(1–27) (10 µg/ml) in blocking buffer for 30 min and washing with Tris-buffered saline (TBS) buffer containing 0.1% Tween 20, the array was developed with Cy5-streptavidin or horse-radish peroxidase (HRP)-streptavidin in blocking buffer. Scanning or chemiluminescence imaging at the appropriate wavelength showed the signal intensity as a single measurement for each peptide and the intensity of each fluorescent spot on the scanned or imaged microarray slide was quantified. Each spot-feature was analyzed for total intensity and background intensity and corrected for background. Data shown represent the mean values of corrected mean/median of signal intensities from two or three identical subarrays on each microarray image.

### Lymphocyte chemotaxis assay

Migration assays using the CXCR4-expressing JVM-3 B cell line were performed in a transmigration well as previously described^32^ using the following modifications. Briefly, JVM-3 B cells were sub-cultured and transferred to media without FBS. A migration assay was performed using 24-well format Transwell membranes (Sigma-Corning; 5 µm pore size) containing 1 × 10^6^ JVM-3 cells in the upper chamber. 8 nM or 16 nM of WT-MIF and 8 nM, 16 nM, or 32 nM of R87A-L88A-R89A-MIF was added to the lower chamber as chemoattractant. As positive control, 8 nM CXCL12 was used. Wells without chemoattractant served as negative control and were used to normalize migration effects to ‘chemotactic index’ (number of migrated cells in the presence of chemoattractant divided by the number of migrated cells in the absence of the chemoattractant) – as described previously^30^. In the inhibition assay, 10 nM, 100 nM, or 1 µM of the RLR-containing MIF peptides 76–90 (YSKLLCGLLAERLRI) or 86–100 (ERLRISPDRVYINYY) were pre-incubated with WT-MIF for 30 min prior to the migration process and also added to the upper chamber. Cells were allowed to migrate for 24 h. After migration, cells in the lower chamber were counted using Countbright absolute counting beads (Invitrogen) using a FACSVerse flow cytometer (BD Biosciences, Heidelberg, Germany).

### Circular dichroism spectroscopy

Far-UV circular dichroism (CD) spectra were recorded in a Jasco 700 CD spectropolarimeter (Jasco Labor- u. Datentechnik GmbH, Groß-Umstadt, Germany). Scans were recorded at 25 °C between 195 and 250 nm as an average of three scans and smoothed to obtain the final data. Spectra were collected at 1.0 nm intervals with a bandwidth of 1 nm in a buffer containing 10 mM sodium phosphate, pH 7.2. Spectra of WT-MIF and R87A-L88A-R89A-MIF were measured at concentrations of 1, 2.5, and 5 µM and were recorded in a 1 cm quartz cuvette. CD spectra are presented as a plot of mean residue ellipticities. Dynode voltage values generally were below 800 and did not interfere with CD measurements. Secondary structure fractions were quantitated by the Dichroweb online software webtool by deconvolutions of CD spectra using ContinLL at DichroWeb and the reference spectra set 7^43,44^.

### Fluorescence spectroscopy

Fluorescence spectroscopy titrations of R87A-L88A-R89A-MIF with the N-terminal peptide of CXCR4 were performed as previously described for WT-MIF^35^. Briefly, titrations were recorded in quartz cuvettes in a JASCO FP-6500 fluorescence spectrophotometer. MIF-CXCR4 interactions were probed by titrating CXCR4 peptide(1–27) against Alexa Fluor-488- R87A-L88A-R89A-MIF. The triple mutant MIF was applied at a concentration of 6.5 nM in 20 mM sodium phosphate buffer, pH 7.2, and the peptide was added at ratios of 1:0.76, 1:1.52, 1:7.63, 1:76.3, and 1:763 in the same buffer. Changes in Alexa Fluor-488 emission were recorded between 500 and 600 nm wavelength.

### Structural models

Three-dimensional structures of human MIF, human CXCL12, and human β-defensin-3 (HBD3) were visualized using the PyMOL Molecular Graphics System, version 1.8.2.2 (Schrödinger, LLC, New York). Surface charge distributions were calculated using PyMOL’s protein contact potential function. The structures were modeled according to the Protein Data Bank (PDB) file for human MIF (PDB identifier: 3DJH), human CXCL12 (PDB identifier: 1SDF), HBD3 (3KJ6), human CXCR4 (Chain B of 2N55), or our molecular docking results. In the structure file of the N-terminus of CXCR4, there are two additional amino acids – glycine and serine – prior to the coding sequence. For clarity in the visualization, these residues were removed before producing the images.

### Molecular docking

For molecular docking simulations of MIF with the N-terminus of CXCR4, we used the PatchDock + FireDock framework. The online tool PatchDock (Beta 1.3 version) for rigid body docking was used with complex type set to default and a clustering root-mean-square deviation (RMSD) of 4.0 Å^78^. Monomeric MIF (chain A of the human MIF structure file 3DJH) was used as a ‘ligand’, the N-terminal region (residues 1–38) of CXCR4 (chain B of the structure file 2N55) as the ‘receptor’. The 1000 best solutions obtained by PatchDock were then submitted to FireDock for refinement by introducing flexibility in the docking process, and rescoring according to free energy calculations^79,80^. Out of the calculated complexes, the highest-ranking solution was chosen for further analysis and visualization.

### Statistical analysis

Data are expressed as means ± SD. Student’s t-tests (two-sided, unpaired) was performed to compare experimental groups. Differences with a value of *p* < 0.05 were considered statistically significant.

## Electronic supplementary material

Supplemental Information
