# Supplementary material for: Identification of an Arg-Leu-Arg tripeptide that contributes to the binding interface between the cytokine MIF and the chemokine receptor CXCR4
Source: Sci Rep. 2018 Mar 26;8:5171. doi: 10.1038/s41598-018-23554-5 (PMC5979958; doi:10.1038/s41598-018-23554-5)
Supplement: Supplementary file 1 — Supplemental Information [file 41598_2018_23554_MOESM1_ESM.pdf]

Identification of an Arg-Leu-Arg tripeptide that contributes to the binding interface between the cytokine MIF and the chemokine receptor CXCR4

Michael Lacy[1,§], Christos Kontos[2,§], Markus Brandhofer[1,§], Kathleen Hille[2], Sabine Gröning[3], Dzmitry Sinitski[1], Priscila Bourilhon[1], Eric Rosenberg[4], Christine Krammer[1], Tharshika Thavayogarajah[1], Georgios Pantouris[4], Maria Bakou[2], Christian Weber[5,6,7], Elias Lolis[4], Jürgen Bernhagen[1,6,8,]*, Aphrodite Kapurniotu[2,]*

[1]Department of Vascular Biology, Institute for Stroke and Dementia Research, Klinikum der Universität München, Ludwig-Maximilians-University of Munich, Feodor-Lynen-Str. 17, D-81377 Munich, Germany; [2]Division of Peptide Biochemistry, Technische Universität München, D-85354 Freising-Weihenstephan, Germany; [3]Department of Anaesthesiology, RWTH Aachen University Hospital, D-52074 Aachen, Germany; [4]Department of Pharmacology, Yale University School of Medicine, 333 Cedar Street, New Haven, CT, USA; [5]Institute for Cardiovascular Prevention, Klinikum der Universität München, Ludwig-Maximilians-University of Munich, Pettenkofer Str. 8, D-80336 Munich, Germany; [6]Munich Heart Alliance, D-80802 Munich, Germany; [7]Cardiovascular Research Institute Maastricht, Maastricht University, 6229 Maastricht, The Netherlands; [8]Munich Cluster for Systems Neurology, D-81377 Munich, Germany.

[§]Equally contributing first authors

*Corresponding authors:

Professor Dr. Aphrodite Kapurniotu, Tel.: +49 8161 713542; Fax: +49 8161 713298; *E-mail address*: akapurniotu@wzw.tum.de

Professor Dr. Jürgen Bernhagen, Tel.: +49 89 4400 46151; Fax: +49 89 4400 46148; *E-mail address*: juergen.bernhagen@med.uni-muenchen.de

Table of contents

- Supplemental figures
- Supplemental tables
- Full-length gels/blots

a

75-90	SYSKLLCGLLAE**RLR**I
scr1	SLALKLEYISRLGRLC
scr2	SLCSIRLALKYRLGLE
scr3	SRRGLLLLSAEYLCKI
scr4	SLAYSRLGIEKLLLRC
scr5	SRLCLALKLEYISRLG

b

MIF (75-90) *versus* scrambled

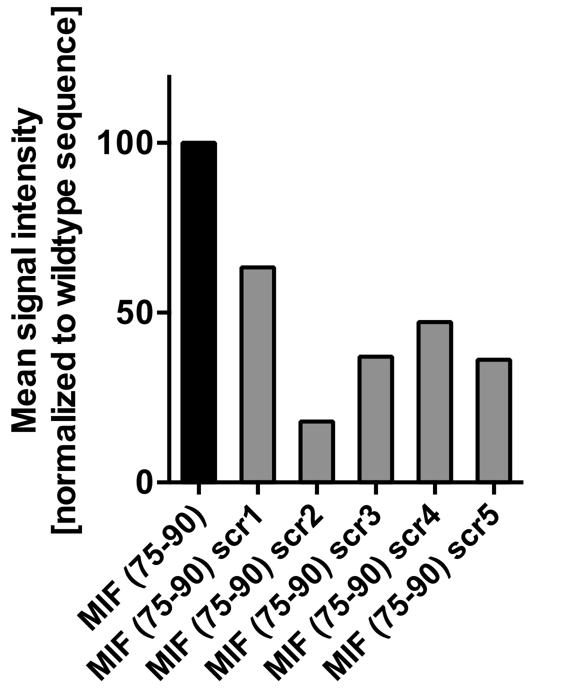

Supplementary Figure 1: Randomization of RLR-containing MIF sequence 75-90 leads to reduction in binding to CXCR4(1-27). Binding of peptide sequences to biotin-CXCR4(1-27) was analyzed by the micro slide-based peptide array technology (Intavis). Sequence 75-90 was randomized by the 'shuffle protein' method (www.bioinformatics.org) to generate the randomized peptides scrambled 1 (scr1), scr2, scr3, scr4, and scr5 1. Bars represent mean signal values of duplicate slides (mean signal intensity) relative to the binding signal of the wildtype peptide MIF(75-90).

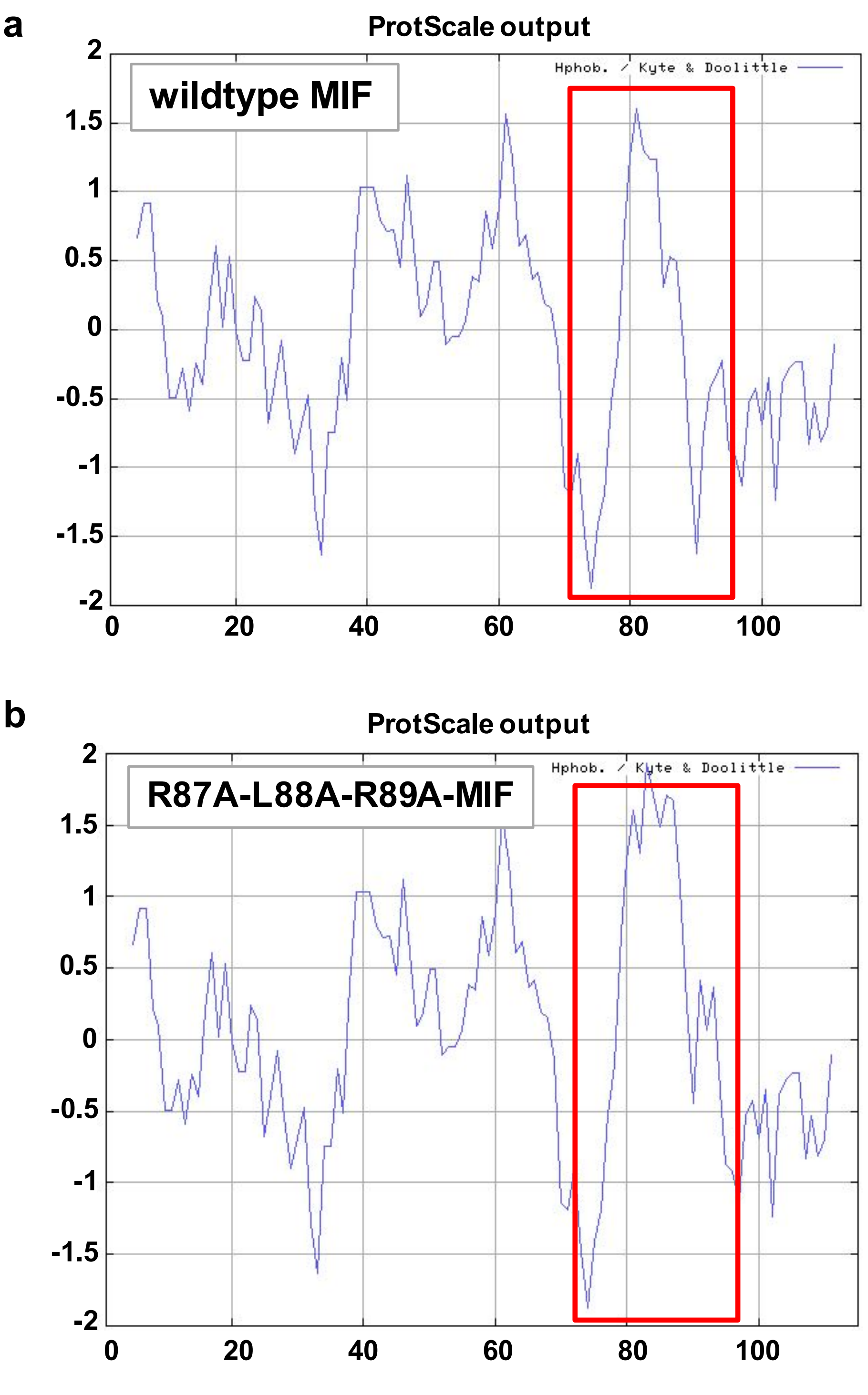

Supplementary Figure 2: Comparison of the hydrophobicity of R87A-L88A-R89A-MIF and wild-type MIF (WT-MIF) using GRAVY. The grand average of hydropathy (GRAVY) index is based on the Kyte & Doolittle algorithm and involved plotting the relative hydrophobicity (y-axis) *versus* the MIF or RLR mutant sequence (x-axis). The sequence region around RLR(87-89) is framed in red.

a

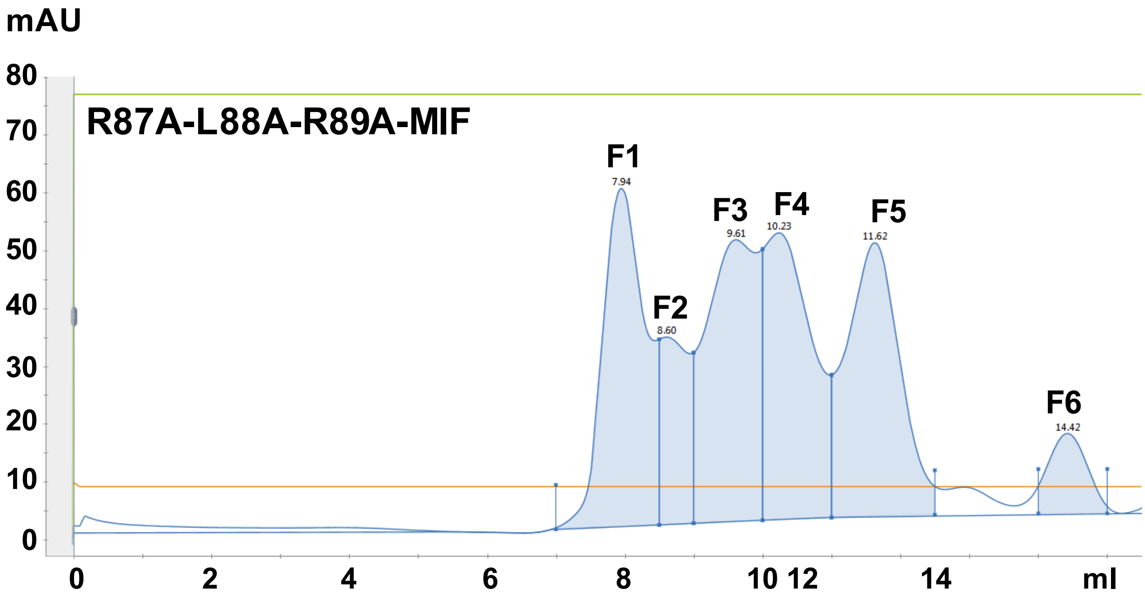

Supplementary Figure 3: Purification of R87A-L88A-R89A-MIF by size exclusion chromatography. Representative chromatogram of a Superdex 75 gel filtration run of the RLR mutant. Fractions F1 through F6 were collected and analyzed for RLR mutant content (see **Fig. 2a-b** for SDS-PAGE and Western blot analysis). Fractions F5 contained most of the enriched mutant protein. F3 and 4 contained minor portions of RLR mutant, which tended to form high-molecular weight oligomers. The chromatogram shown is plotted as UV absorption at 280 nm (in [mAU]; blue line) over the elution volume in [ml]. The column was run at 0.75 ml/min. The green line indicates that elution was done with one buffer (100% B; no gradient); orange line indicates conductivity, which was read for control. Small font numbers next to the fraction captions F1-F6 indicate the exact elution volumes of the peaks.

Supplementary Figure 4; Lacy et al., revised 2018

a

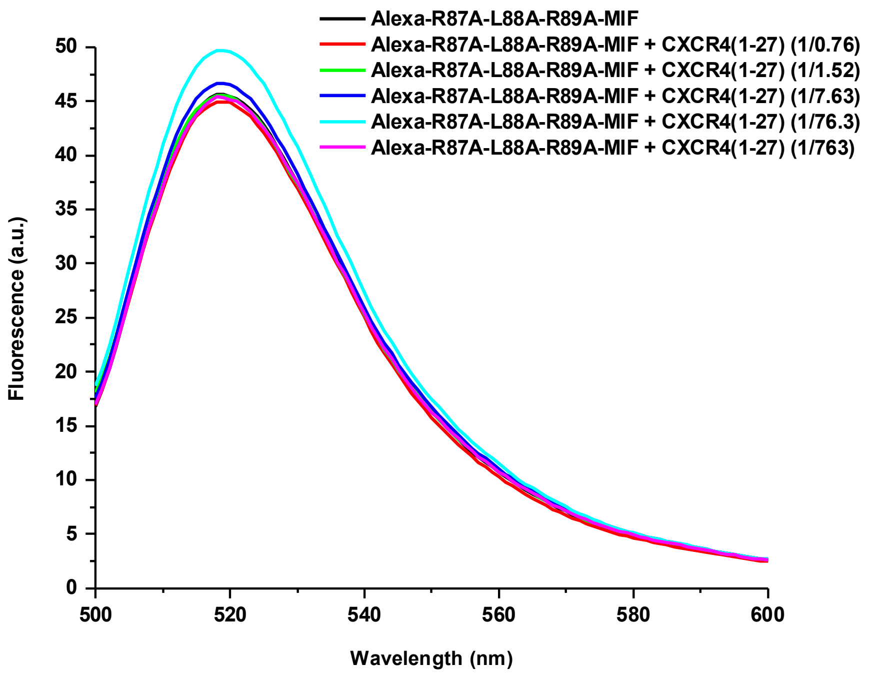

b

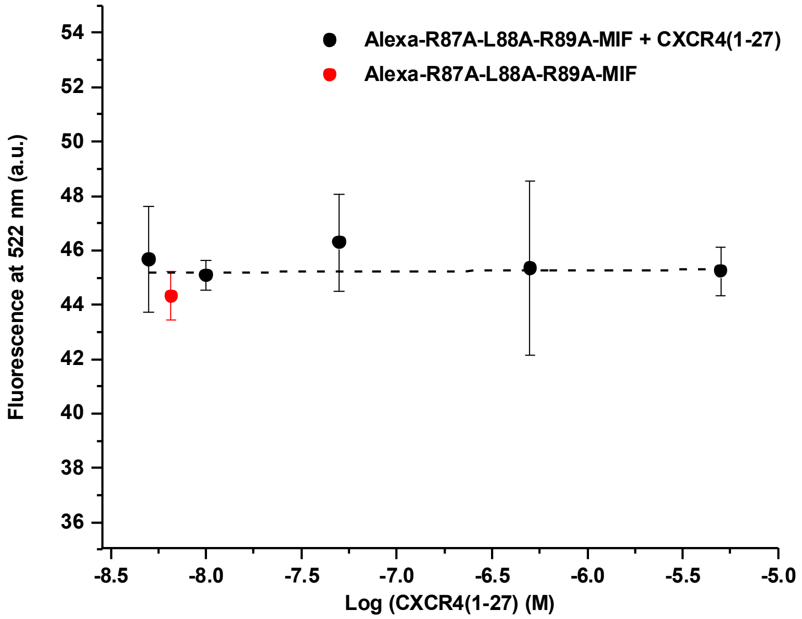

Supplementary Figure 4: The RLR triple alanine mutant does not bind to CXCR4(1-27). (**a**) Fluorescence spectroscopic titration of Alexa-488-R87A-L88A-R89A-MIF with increasing ratios of CXCR4(1-27). The solution mixture of CXCR4(1–27) peptide with Alexa-488-R87A-L88A-R89A-MIF does not evoke a conformational change in the mutant protein as indicated by a lack of change in fluorescence. Peptide was added at molar ratios of 1:0.76, 1:1.52, 1:7.63, 1:76.3, 1:763 and spectra were recorded between wavelengths 500 and 600 nm (arbitrary units, a.u.). (**b**) Concentration dependence of the CXCR4 peptide plotted against the fluorescence change at 522 nm. The horizontal dashed line is a fit through the data points and indicates no conformational change in the mutant protein.

Supplementary Figure 5; Lacy et al., revised 2018

a

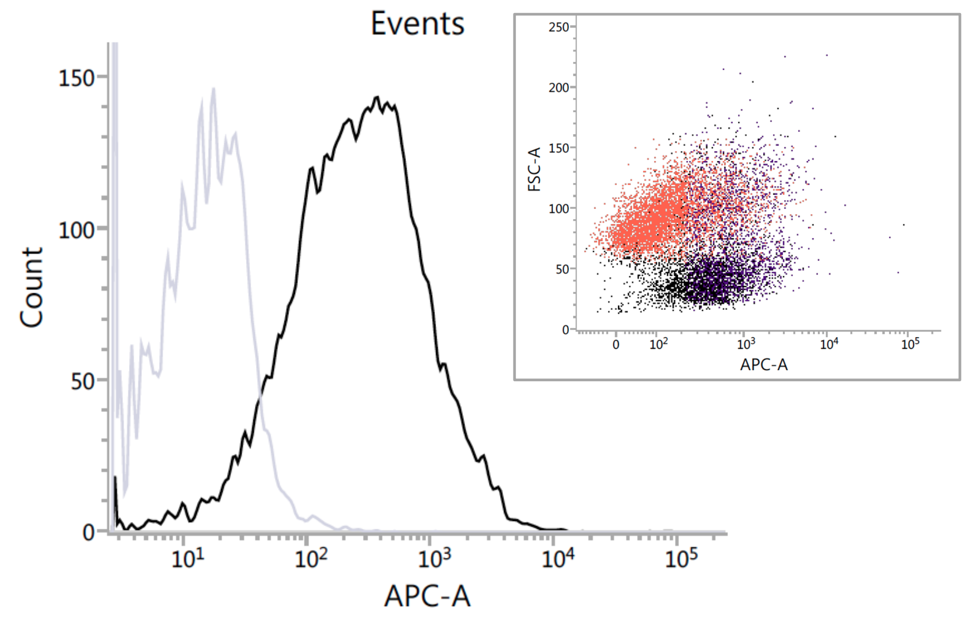

b

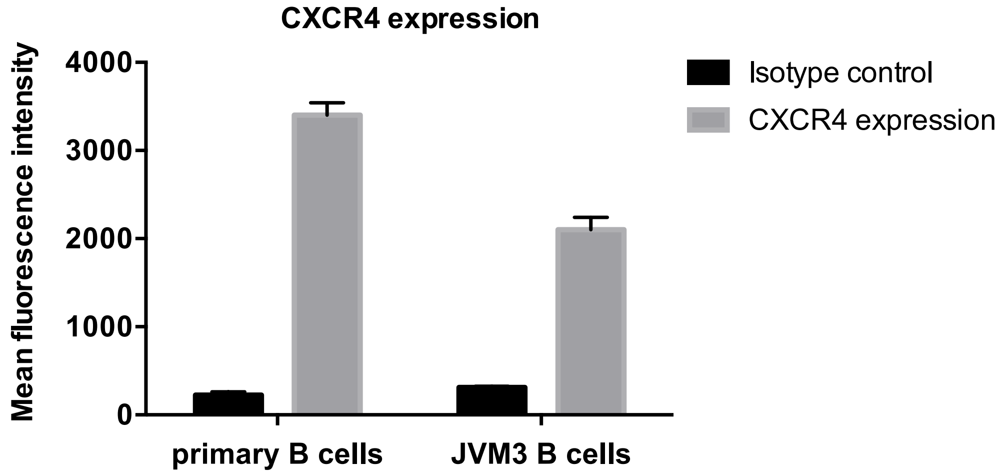

Supplementary Figure 5: JVM-3 B lymphocytes express marked levels of CXCR4. Flow cytometry analysis of JVM-3 cells. (**a**) FACS histogram showing cell counts against staining for surface CXCR4 in JVM-3 by allophycocyanin (APC)-A label (black curve). Grey curves indicates the non-specific signal using isotype IgG control instead of anti-CXCR4-antibody. Inset, dot-plot of forward scatter (FSC-A) versus APC-A signal. (**b**) Comparison of CXCR4 expression (plotted as mean fluorescence intensity (MFI)) in JVM-3 B cells and primary human B cells isolated via MACS selection from peripheral blood mononuclear cells (PBMCs) of healthy volunteers. Specific signals using anti-CXCR4 antibody versus controls applying isotype immunoglobulin (IgG2a). Triplicate measurements ± SD.

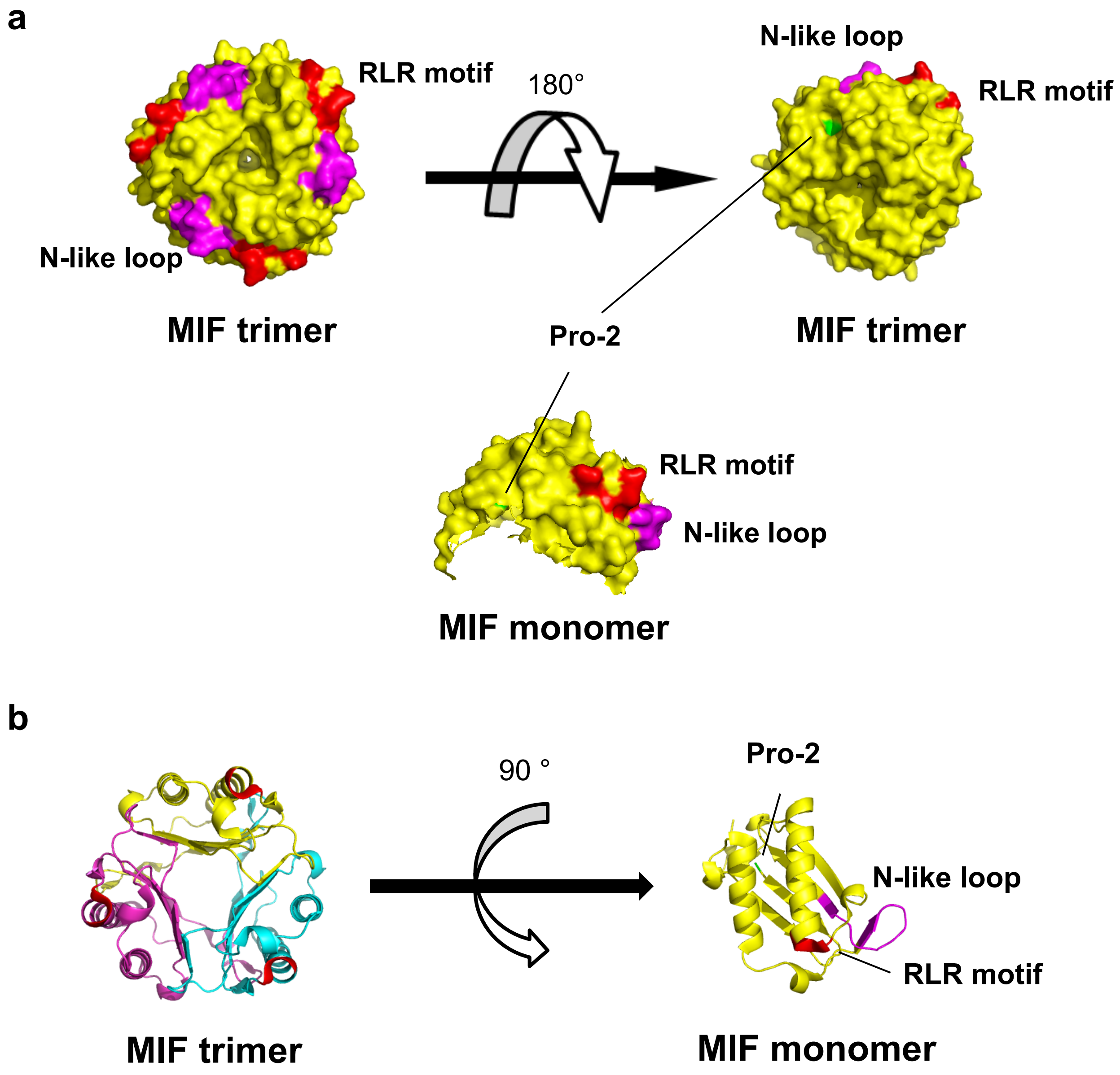

Supplementary Figure 6: Structure models of the human MIF monomer and trimer, highlighting the interaction motifs implicated in CXCR4 binding. (**a**) Surface structure model. The MIF motifs implicated in CXCR4 binding, i.e. N-like loop (pink), RLR motif (red), and Pro-2- residue (green) are highlighted. (**b**) Ribbon structures. Same color code and captions as in (a). Protein structures were produced/visualized with PyMOL.

Supplementary Table 1: Purification and enrichment of R87A-L88A-R89A-MIF compared to wildtype MIF. Overview of the purification scheme.

	**Total protein in bacterial lysate [mg] / % purity**	**Purification by anion exchange column [mg] / % purity**	**Secondary purification step (C8 or SEC*) [mg] / % purity**
WT-MIF	96.9 / 10-20%	20.5 / 80-85%	3.2 / 95-98%
R87A-L88A-R89A-MIF	87.7 / 10-20%	4.2 / 50-60%	1.4 / >90%

For each step, total protein in [mg] (left number) and enrichment factor in % purity of total protein (right number) is given. C8, last step of the WT-MIF purification scheme via C8 reverse phase column that has been established previously to result in 95-98% pure MIF protein in endotoxin-free form (see ref. 29); SEC, size exclusion chromatography, last step of the R87A-L88A-R89A-MIF purification procedure (see Methods).

Supplementary Table 2: Circular dichroism (CD) spectropolarimetry shows that the folding and secondary structure profile of R87A-L88A-R89A-MIF is similar to that of WT-MIF. Quantification of the secondary structure contents of the CD spectra shown in **Fig. 2b**.

	**α-helix**	**β-strand**	**β-strand / β-turn**	**Unordered**	**NRMSD**
WT-MIF	44.7	27.8	48.6	6.8	0.090
R87A-L88A-R89A-MIF	32.3	21.0	39.8	28.7	0.044

Conformations in the CD spectra were measured as mean residue ellipticity versus the wavelength in the far-UV range. Quantification of the secondary structure fractions of WT-MIF and R87A-L88A-R89A-MIF as analyzed by the Dichroweb online software webtool. Secondary structure contents (%) were calculated by deconvolutions of CD spectra which was performed using ContinLL at DichroWeb and the reference spectra set 7. NRMSD (normalized root mean square deviation) of fits.

Full-length gel of Figure 2a

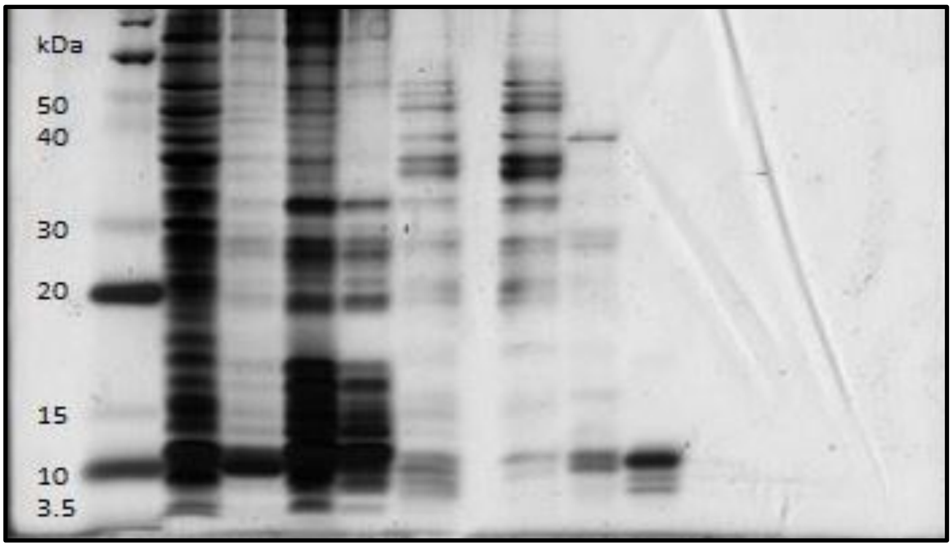

The original full gel underlying Figure 2 is shown above. To produce Figure 2a, the following 3 lanes on the left hand side of the gel were not shown: i) molecular weight markers (lane 1); ii) non-induced bacteria (not concentration-adjusted); iii) induced bacteria (not concentration-adjusted). Lane 4 equals the first lane in Figure 2a, i.e. the total lysate lane. The right-hand side of the gel also was cropped (empty lanes).

Full-length blot of Figure 2b

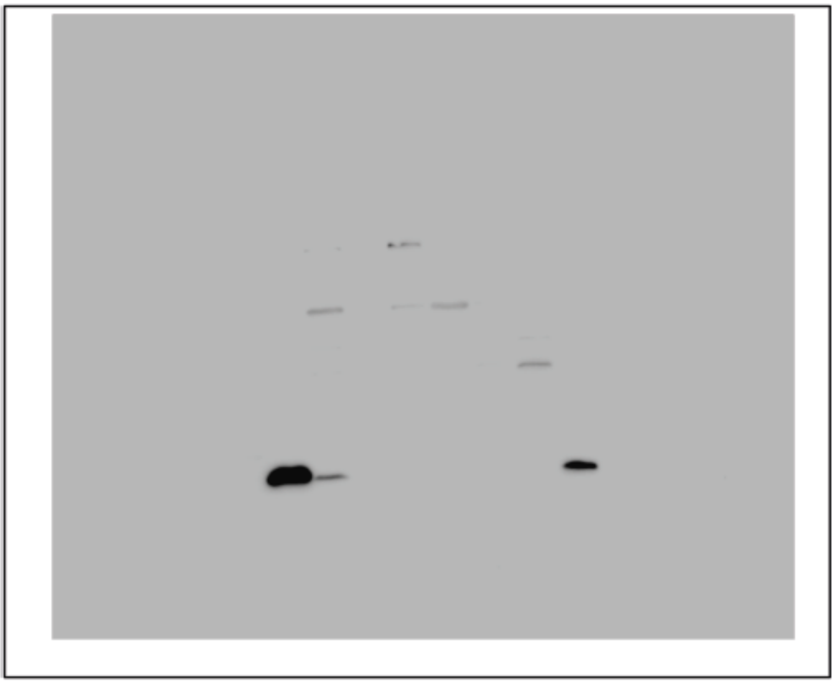

To produce Figure 2b, the blot image was cropped on the left and right side (empty space).
